# Genomic and molecular features distinguish young adult cancer from later-onset cancer

**DOI:** 10.1016/j.celrep.2021.110005

**Published:** 2021-11-16

**Authors:** William Lee, Zishan Wang, Miriam Saffern, Tomi Jun, Kuan-lin Huang

**Affiliations:** 1 Department of Genetics and Genomic Sciences, Center for Transformative Disease Modeling, Tisch Cancer Institute, Icahn Institute for Data Science and Genomic Technology, Icahn School of Medicine at Mount Sinai, New York, NY 10029, USA; 2 Precision Immunology Institute, Icahn School of Medicine at Mount Sinai, New York, NY 10029, USA; 3 Division of Hematology and Medical Oncology, Tisch Cancer Institute, Icahn School of Medicine at Mount Sinai, New York, NY 10029, USA; 4 Lead contact

## Abstract

Young adult cancer has increased in incidence worldwide, but its molecular etiologies remain unclear. We systematically characterize genomic profiles of young adult tumors with ages of onset ≤50 years and compare them to later-onset tumors using over 6,000 cases across 14 cancer types. While young adult tumors generally show lower mutation burdens and comparable copy-number variation rates compared to later-onset cases, they are enriched for multiple driver mutations and copy-number alterations in subtype-specific contexts. Characterization of tumor immune microenvironments reveals pan-cancer patterns of elevated TGF-β response/dendritic cells and lower IFN-γ response/macrophages relative to later-onset tumors, corresponding to age-related responses to immunotherapy in several cancer types. Finally, we identify prevalent clinically actionable events that disproportionally affect young adult or later-onset cases. The resulting catalog of age-related molecular drivers can guide precision diagnostics and treatments for young adult cancer.

## In brief

In recent years, the incidence of multiple cancer types has risen among young adults under 50 years, but whether they show specific molecular etiology remains unclear. Lee et al. characterize the genomic and tumor microenvironment features distinguishing young adults from later-onset cancer and potential treatment implications.

## Graphical abstract

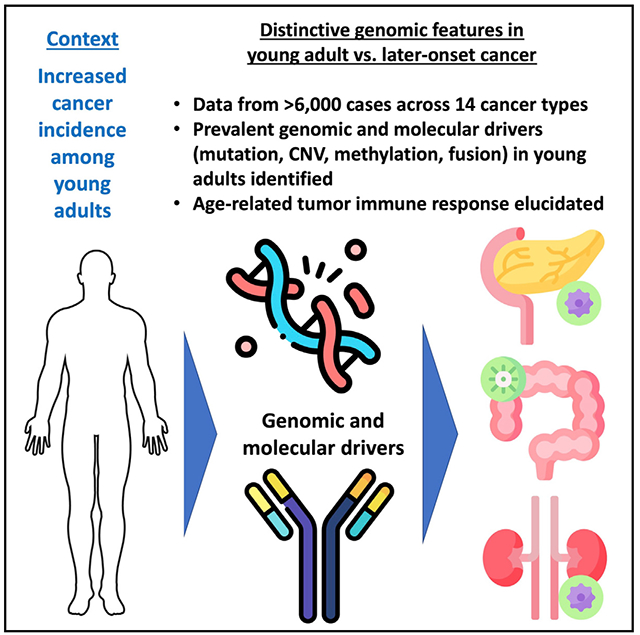

## INTRODUCTION

In the US and multiple countries, cancer incidence and mortality have steadily declined in adults over the age of 50 (Bray et al., 2018; Siegel et al., 2020). Alarmingly, the overall decline is coupled with a recent rise in the incidence of various cancer types among young adults, including colorectal, endometrial, gallbladder, multiple myeloma, pancreatic, and renal cancer, for individuals between 15 and 50 years old in the US (Sung et al., 2019) and worldwide (Gupta et al., 2020). However, existing genomic and molecular studies of adult cancers comprise mainly later-onset individuals (Campbell et al., 2020; Sanchez-Vega et al., 2018; Zehir et al., 2017), where alterations that are differentially prevalent in young adult cancers may be diluted. Comprehensive genomic analyses of young adult tumors are required to reveal their molecular underpinnings, which will in turn improve targeted diagnostics and treatments for this understudied population.

The molecular etiologies of young adult cancer are likely distinct from those of pediatric or later-onset adult cancer (Bleyer et al., 2008). Early-onset colorectal cancer is known to be associated with germline mutations in mismatch repair (MMR) genes (Lynch syndrome) (Lynch et al., 2009; Pearlman et al., 2017), and germline mutations disrupting *ATM, CHEK2, BRCA1/2, CDKN2A*, and *PALB2* may also contribute to early-onset colorectal carcinogenesis (Pearlman et al., 2017). Similarly, multiple studies have confirmed a higher prevalence of germline mutations in *BRCA1/2* and *TP53* in early-onset breast cancer (Gómez-Flores-Ramos et al., 2017; Packwood et al., 2019; Peto et al., 1999). However, while studies have identified various germline predisposing variants associated with young adult cancer, large-scale investigation of their somatic genomic and molecular profiles is scarce.

In this study, we conducted a comprehensive young adult pan-cancer genomic analysis using data from 1,757 young adult and 3,608 later-onset cases across 14 different cancer types in The Cancer Genome Atlas (TCGA), verified by analyses using non-overlapping cases in the International Cancer Genome Consortium (ICGC). We systematically identified somatic mutation, copy-number variation (CNV), DNA methylation, and immune features in young adult cohorts and compared them to those of later-onset cancer. Analyses of the tumor immune microenvironment revealed signaling and cellular features distinguishing young adults from older adults. Additionally, we identified treatment response-associated mutations and amplifications most frequently appearing in young adults, which may help to advance precision oncology applications for young adult cancer.

## RESULTS

### Cohort characteristics and mutation rates

**Figure 1.**
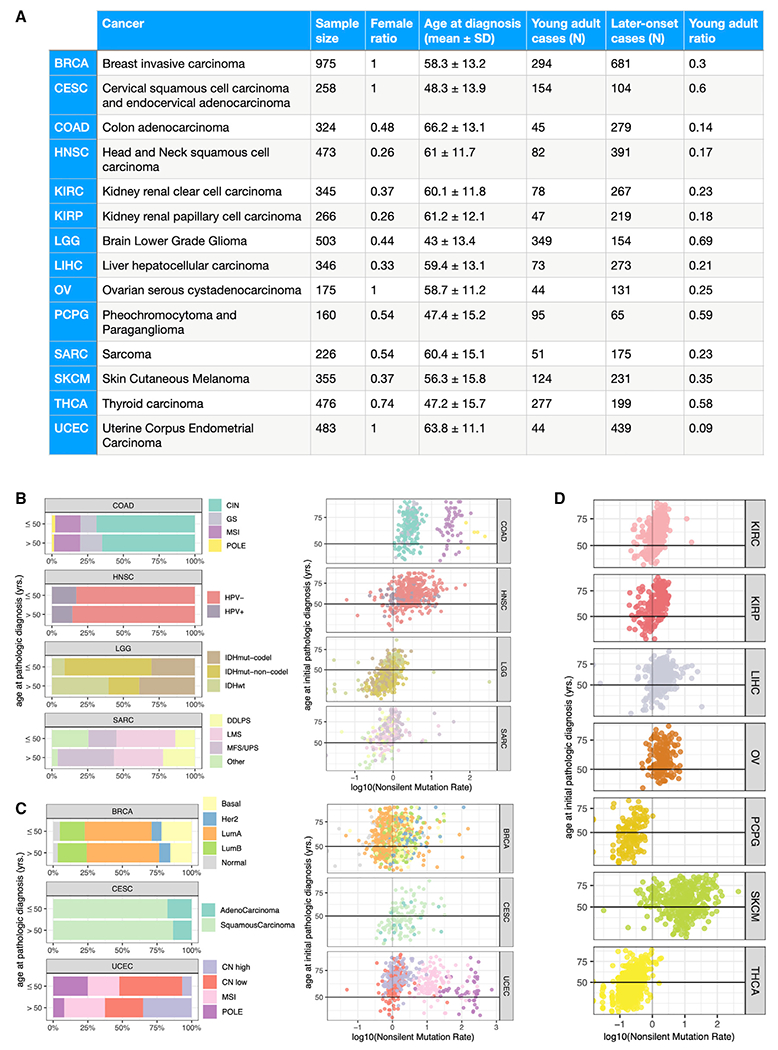
Characterizing 5,365 adult cancers of the TCGA PanCanAtlas cohort (A) Attributes of the 5,365 cases (1,757 young adult and 3,608 later-onset) across 14 cancer types, including TCGA abbreviation of the cancer type, gender ratio, mean age at diagnosis, and young adult ratio. (B) Subtype percentages between young adult and later-onset cases in non-female-specific cancer types. Log10-transformed nonsilent mutation rates for unique individuals in each cancer type are colored by subtype. (C) Subtype percentages between young adult and later-onset cases in female-specific cancer types. Log10-transformed nonsilent mutation rates for unique individuals in each cancer type are colored by subtype. (D) Log10-transformed nonsilent mutation rates for unique individuals in cancer types without subtype information are distinguished by PanCanAtlas colors. See also Figure S1.

We utilized TCGA cohort to characterize the genomic and molecular profiles of young adult cancer. Matching the young adult population defined by epidemiological studies that show increased cancer rates (Sung et al., 2019), we defined young adult cases as those with an age at initial pathologic diagnosis ≤50 years, while subjects >50 were classified as later-onset cases. Of the 33 cancer types in TCGA, 14 included ≥40 unique, pass quality-control cases in both the young adult and later-onset cohorts and were retained for subsequent analyses (STAR Methods), amounting to a total sample size of 1,757 young adult and 3,608 later-onset cases. While several cancer cohorts afford sufficient young adult cases for analysis, their minor representations across the majority of these cancer types (except for LGG [brain lower grade glioma], CESC [cervical squamous cell carcinoma and endocervical adenocarcinoma], PCPG [pheochromocytoma and paraganglioma], and THCA [thyroid carcinoma]) highlight the need for tailored investigations (Figure 1A). The ICGC cohort was further used to validate the age-related genomic driver events (Campbell et al., 2020). After retaining only cases (1) with age at diagnosis data, (2) matched to one of the 14 analyzed TCGA cancer types, and (3) not overlapped with TCGA cases, the ICGC validation dataset contained 127 young adult and 515 later-onset samples (STAR Methods).

Of the 14 selected cancer types, seven had available, defined subtypes (Figures 1B and 1C). We first assessed whether the subtypes enriched in young adults in TCGA were concordant with previous reports to ensure the young adult cancer populations studied herein were representative. A significantly lower percentage of young adult LGG as compared to later-onset LGG were of the wild-type *IDH1* and *IDH2* subtype (Fisher’s exact test, IDHwt, false discovery rate [FDR] = 7.62E-15), in agreement with a previous report (Brat et al., 2015), while a nearly proportionally greater percentage were of the mutated *IDH*, no 1p/19q codeletion subtype (IDHmut-non-codel, FDR = 3.86E-16) (Figure 1B). We also observed a suggestive association between later-onset LGG and the IDHmut-codel subtype (FDR = 0.064). A significantly lower percentage of young adult SARC (sarcoma) cases were of the MFS/UPS subtype (myxofibrosarcoma/undifferentiated pleomorphic sarcoma, FDR = 0.024, Figure 1B), consistent with previous findings (Look Hong et al., 2013; Sessa et al., 2019). Young adult BRCA (breast invasive carcinoma) was suggestively associated with the basal subtype (FDR = 0.105, Figure 1C), affirming previous findings of basal breast cancer’s associations with earlier ages of onset (Bertucci et al., 2012). Additionally, significantly higher percentages of young adult UCEC (uterine corpus endometrial carcinoma) were of the POLE (*POLE*-ultramutated, FDR = 2.55E-3) and CN low (copy number low, FDR = 0.030) subtypes, while a significantly lower percentage were of the CN high subtype (copy number high, FDR = 1.46E-4) (Figure 1C).

We next examine the mutation rate, which is known to show age dependency (Gao et al., 2016; Risques and Kennedy, 2018). As expected, higher silent and nonsilent mutation rates were found in later-onset cases across all cancer types, except LGG and UCEC (Figures 1B–1D and S1A–S1D). In CESC, KIRC (kidney renal clear cell carcinoma), KIRP (kidney renal papillary cell carcinoma), PCPG, and THCA, the positive associations between age and both mutation rates reached significance (FDR <4.81E-3). Surprisingly, young adult UCEC showed both significantly higher silent (FDR = 4.92E-5) and nonsilent (FDR = 5.24E-5) mutation rates (Figure S1A). A closer examination revealed higher mutation rates in the POLE subtype prevalent in young adult UCEC (Figure 1C), suggesting cancer subtype and onset age need to be jointly considered in estimations of mutation rate in selected cancer types.

### Somatic mutation landscapes

**Figure 2.**
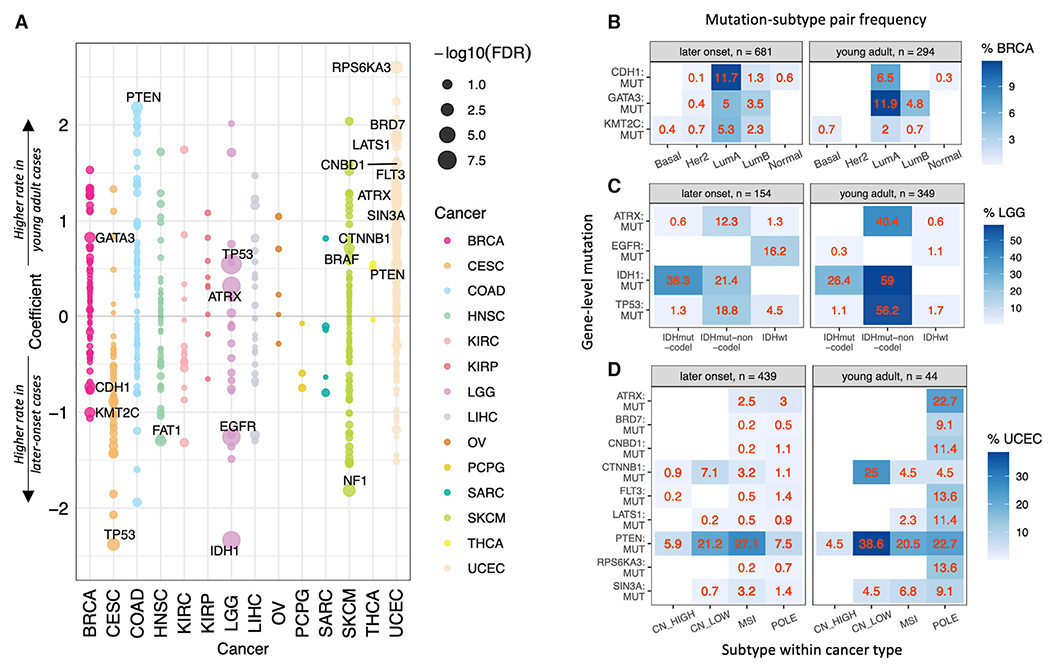
Somatic mutations between young adult and later-onset tumors (A) Somatic mutations associated with young adult versus later-onset cancer cohorts. For each gene-level mutation, a coefficient >0 represents a higher rate in young adult cases, while a coefficient <0 represents a higher rate in later-onset cases. Significant mutations (FDR <0.05) are labeled. (B) Percentages of young adult versus later-onset BRCA cases presenting mutation-subtype pairs. (C) Percentages of young adult versus later-onset LGG cases presenting mutation-subtype pairs. (D) Percentages of young adult versus later-onset UCEC cases presenting mutation-subtype pairs. See also Figure S2 and Table S1.

We conducted a multivariate regression analysis to identify cancer driver genes showing different mutation rates of likely driver mutations in young adult compared to later-onset cancer, corrected for covariates including subtype (when available), gender, and genetic principal components that represent population structures (STAR Methods). We identified 21 significant (FDR <0.05) associations across 18 genes (Figure 2A; Table S1). While overall mutation rates are lower in young adult cases, multiple of the significant associations were enriched mutations in young adults. *GATA3* somatic mutations were significantly associated with young adult cases in BRCA (FDR = 0.036). Somatic mutations in *PTEN* were significantly associated with young adult COAD (colon adenocarcinoma) (FDR = 0.022) and UCEC (FDR = 0.028). *ATRX* mutations were significantly associated with both young adult LGG (FDR = 0.022) and UCEC (FDR = 0.027) (Figure 2A). In addition, somatic mutations of *BRD7, CNBD1, CTNNB1, FLT3, LATS1, RPS6KA3*, and *SIN3A* (FDR ≤0.045) were each significantly associated with young adult UCEC. We also found *TP53* and *BRAF* somatic mutations to be significantly associated with young adult LGG (FDR = 3.25E-10) and SKCM (skin cutaneous melanoma) (FDR = 0.045), respectively (Figure 2A). Conversely, somatic mutations of *TP53* (FDR = 5.09E-3), *FAT1* (FDR = 0.027), and *NF1* (FDR = 5.71E-3) were significantly associated with later-onset CESC, HNSC (head and neck squamous cell carcinoma), and SKCM, respectively. Additionally, somatic mutations in *KMT2C* (FDR = 0.046) and *CDH1* (FDR = 0.045) were found to be significantly associated with later-onset BRCA, while those in *EGFR* (FDR = 1.34E-7) and *IDH1* (FDR = 1.34E-7) were found to be significantly associated with later-onset gliomas (Figure 2A). To ensure robustness, we also conducted analyses treating age as a continuous variable in the multivariate model, again finding that *TP53* and *ATRX* somatic mutations were each associated with decreased age in LGG, while the opposite trend was observed for *IDH1* (FDR <8.96E-4, Figure S2A).

Given that somatic mutations may show specific associations with subtypes, we next compared the rate of mutation-subtype pairs within cancer types to their young adult versus later-onset status using Fisher’s exact tests (STAR Methods), identifying 16 significant and three suggestive associations. We found significant enrichment of *GATA3*-mutated luminal A BRCA in young adults (FDR = 3.11E-3, Figures 2B and S2B), as well as significant enrichment of *TP53, IDH1*, and *ATRX*-mutated IDHmut-non-codel gliomas in the same cohort (FDR ≤ 4.64E-10, Figures 2C and S2C). *ATRX, BRD7, CNBD1, FLT3, LATS1, PTEN, RPS6KA3*, and *SIN3A*-mutated POLE UCEC were each significantly enriched in young adults (FDR ≤ 0.034, Figures 2D and S2D), along with *PTEN*-mutated (FDR = 0.048) and *CTNNB1*-mutated (FDR = 3.89E-3) CN-low UCEC (Figures 2D and S2D). Conversely, *CDH1*-mutated luminal A (FDR = 0.083) and *KMT2C*-mutated luminal A (FDR = 0.122) BRCA was suggestively enriched in later-onset cases (Figures 2B and S2B). We also found significant enrichments of *EGFR*-mutated IDHwt (FDR = 7.03E-10) and *IDH1*-mutated IDHmut-codel gliomas (FDR = 0.020) in later-onset cases (Figures 2C and S2C). Altogether, these analyses demonstrate that multiple recurrent mutations affecting young adult cancers (e.g., *ATRX* in both LGG and UCEC) were distinct from those affecting later-onset cases, and correlated with their subtype distributions.

To adjust for the potential confounding effect of hypermutator phenotypes, we conducted additional multivariate regression analyses within only the non-hypermutated TCGA samples (STAR Methods). Outside of UCEC, all significant somatic hits were rediscovered, except for the association between COAD and *PTEN* (Figure 2A; Figure S2E). In UCEC, however, none of the significant age-related somatic mutations (with the exception of *CTNNB1* mutations) were re-discovered in our non-hypermutator analysis (Figure 2A, Figure S2E). These results were expected given our subtype analyses results showing an association between young adult UCEC and the hypermutator POLE subtype, where these mutations may be enriched (Figure 1C; Figure 2D; Figure S2D). In contrast and notably, *CTNNB1* mutation, while also associated with young adults, was rediscovered, concordant with the finding of enriched *CTNNB1*-mutated, CN-low UCEC in young adults (Figures 2D and S2D).

To validate the age-related somatic driver mutations found in TCGA, we performed an independent multivariate regression analysis on the ICGC dataset (Campbell et al., 2020) in which we removed the overlapping TCGA samples and mapped cancer types to TCGA. The five ICGC cohorts corresponding to TCGA cancer types include Breast-AdenoCA (corresponding to TCGA BRCA), Liver-HCC (corresponding to TCGA LIHC [liver hepatocellular carcinoma]), Ovary-AdenoCA (corresponding to TCGA OV [ovarian serous cystadenocarcinoma]), Kidney-RCC (corresponding to TCGA KIRC/KIRP), and Skin-Melanoma (corresponding to TCGA SKCM) (Figure S2F). In the ICGC cohort, *RPS6KA3* mutation is also associated with young adult Liver-HCC (p = 0.013, Figure S2F), in addition to its TCGA-identified association with young adult UCEC (Figure 2A). Additionally, ICGC analyses found an association between *PTEN* mutation and young adult Skin-Melanoma (Figure S2F), mirroring the TCGA-identified associations of *PTEN* with both young adult COAD and UCEC (Figure 2A). These findings also suggest possible cross-cancer type associations between young adult tumors and mutations in *RPS6KA3* and *PTEN* (Figure 2A; Figure S2F). Finally, the ICGC validation rediscovered the TCGA-identified associations between mutations in *BRAF* (p = 0.028) and *NF1* (p = 0.021) and young adult and later-onset SKCM, respectively (Figure S2F).

### Copy-number variations

**Figure 3.**
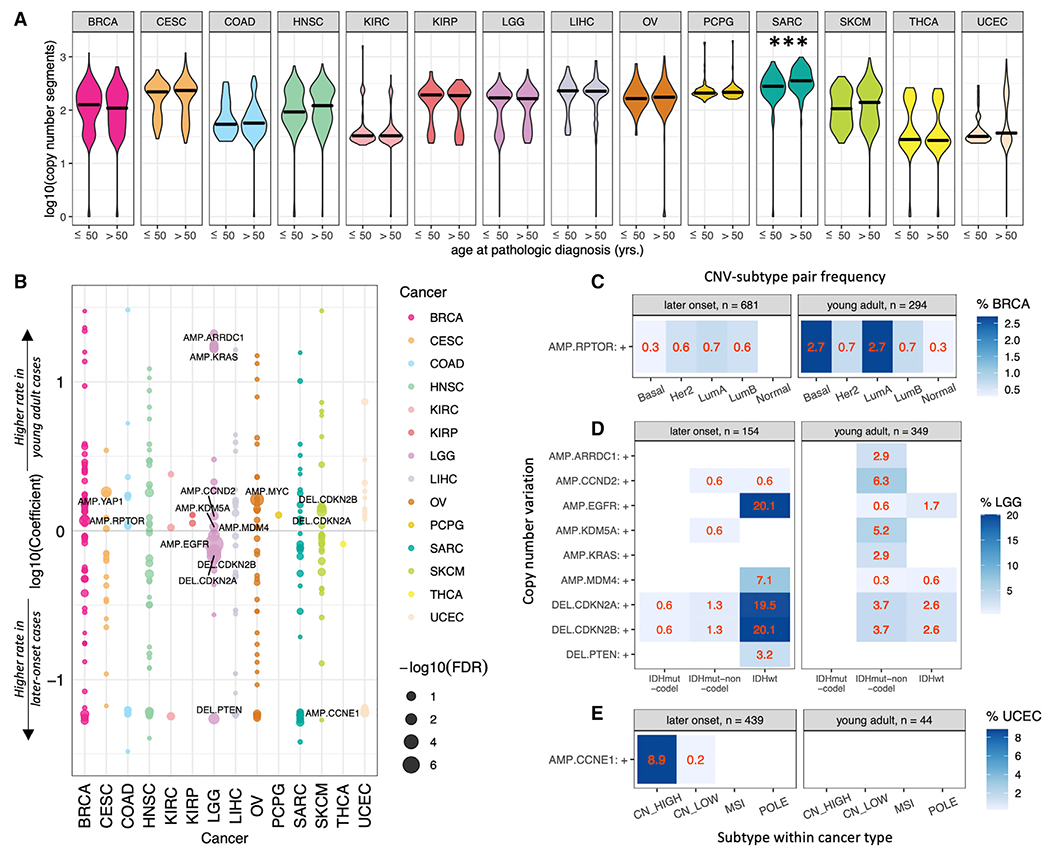
Copy-number variations between young adult and later-onset tumors (A) Log10-transformed summed copy-number segments per sample across the 14 cancer types in young adult versus later onset tumors; black bars designate median values. *FDR ≥0.10 and <0.15, **FDR ≥0.05 and <0.10, and ***FDR <0.05. (B) CNVs associated with young adult versus later-onset cancer cohorts. For each deletion or amplification, a log10-transformed coefficient >0 represents a higher rate young adult cases, while a log10-transformed coefficients <0 represents a higher rate in later-onset cases. Significant (FDR <0.05) and suggestive (FDR <0.15) CNVs are labeled. (C) Percentages of young adult versus later-onset BRCA cases presenting CNV-subtype pairs. (D) Percentages of young adult versus later-onset LGG cases presenting CNV-subtype pairs. (E) Percentages of young adult versus later-onset UCEC cases presenting CNV-subtype pairs. See also Figure S3 and Table S2.

In addition to recurrent mutations, CNVs are critical genomic drivers of tumorigenesis. We first analyzed aneuploidy scores calculated by the summed burden of chromosome arm-level events in each sample (Taylor et al., 2018) (STAR Methods), finding significantly higher aneuploidy among later-onset cases in CESC, KIRC, LGG, OV, SARC, THCA, and UCEC (FDR <0.046, Figures S3A and S3B) and affirming previously reported positive associations between aneuploidy and age (Simonetti et al., 2019). Analyzing the number of copy-number segments per sample (STAR Methods), we found comparable overall CNV rates between young adult and later-onset cohorts across all cancer types except SARC, where later-onset cases showed higher CNV rates (FDR = 0.018, Figures 3A and S3C).

We next analyzed the prioritized driver copy-number deletions and amplifications defined by the PanCanAtlas project (Sanchez-Vega et al., 2018). The same multivariate regression model was applied to identify the gene-level CNVs that showed different rates in young adult compared to later-onset cancer (STAR Methods). We identified eight significant (FDR <0.05) and seven suggestive associations across 13 genes (Figure 3B; Table S2). Notably, the copy-number events exhibiting higher prevalences in young adult cancers were mainly amplifications, including *YAP1* (FDR = 0.027) and *MYC* (FDR = 6.94E-4), significantly associated with young adult CESC and OV, respectively. *RPTOR* amplification was found to be significantly associated with young adult BRCA (FDR = 0.020), while amplifications of *KDM5A, CCND2, KRAS*, and *ARRDC1* were each suggestively associated with young adult gliomas (FDR ≤ 0.148). Additionally, deletions of *CDKN2A* (FDR = 0.060) and *CDKN2B* (FDR = 0.107) were suggestively associated with young adult SKCM (Figure 3B). Copy-number events that showed significant associations with later-onset cases aggregated in LGG: deletions of *PTEN* (FDR = 0.026), *CDKN2A* (FDR = 1.78E-4), and *CDKN2B* (FDR = 1.16E-4) as well as amplifications of *MDM4* (FDR = 0.011) and *EGFR* (FDR = 1.43E-8) were each significantly associated with later-onset gliomas (Figure 3B). In a separate multivariate regression analysis treating age as a continuous variable, we found few associated CNVs including *MYC* amplifications to be suggestively associated with increased age in OV (FDR = 0.121, Figure S3D).

We next compared the rate of CNV-subtype pairs within cancer types to their young adult versus later-onset status (STAR Methods), identifying eight showing significant and four showing suggestive associations. *RPTOR*-amplified basal BRCA was enriched in young adults (FDR = 7.82E-3), while *RPTOR*-amplified luminal A BRCA was suggestively enriched (FDR = 0.068, Figures 3C and S3E). We also identified significant enrichments of both *CCND2*-amplified IDHmut-non-codel (FDR = 0.019) and *KDM5A*-amplified IDHmut-non-codel (FDR = 0.042) gliomas in young adults, along with suggestive enrichments of *KRAS* amplified IDHmut-non-codel (FDR = 0.108) and *ARRDC1* amplified IDHmut-non-codel (FDR = 0.108) gliomas (Figures 3D and S3F). Conversely, later-onset gliomas were associated with the IDHwt subtype paired with *EGFR* or *MDM4* amplification, as well as with *CDKN2A, CDKN2B*, or *PTEN* deletion (FDR <0.015). Suggestive enrichment was found for *CCNE1*-amplified CN-high UCEC in later-onset cases (FDR = 0.076, Figures 3E and S3G).

To validate findings in TCGA, we next performed independent multivariate regression analyses using the ICGC CNV dataset (Gerstung et al., 2020). The TCGA analysis uncovered an association between *CCNE1* CNVs and later-onset UCEC (Figure 3B), and here we find that *CCNE1* CNVs are also associated with later-onset OV (p = 0.020, Figure S3H). The ICGC analysis also validated the TCGA-identified association (Figure 3B) between *CDKN2A* CNV and young adult SKCM (p = 0.028, Figure S3H). These analyses discovered multiple driver copy-number events that show different cancer and subtype prevalences in young adult cancers.

### DNA methylation and fusion events

**Figure 4.**
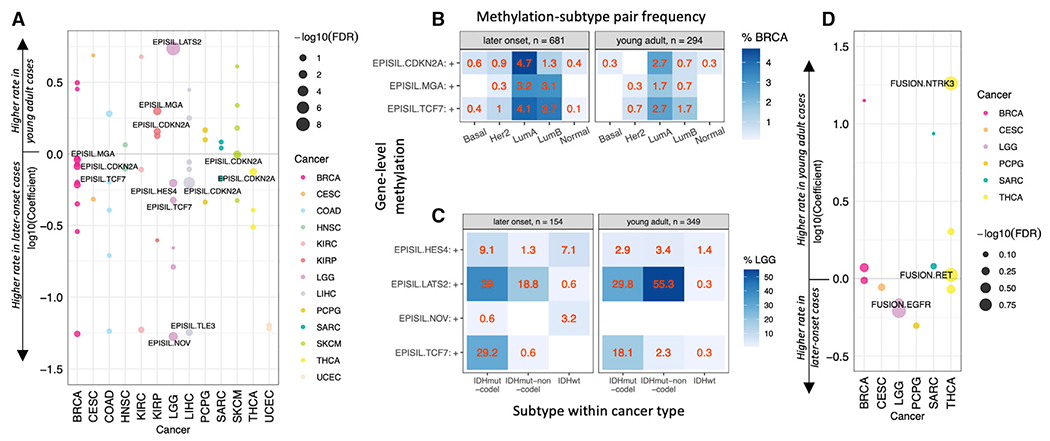
Methylations/fusions between young adult and later-onset tumors (A) Methylations associated with young adult versus later-onset cancer cohorts. For each gene-level methylation, a log10-transformed coefficient >0 represents a higher rate in young adult cases, while a log10-transformed coefficient <0 represents a higher rate in later-onset cases. Significant (FDR <0.05) and suggestive (FDR <0.15) methylations are labeled. (B) Percentages of young adult versus later-onset BRCA cases presenting methylation-subtype pairs. (C) Percentages of young adult versus later-onset LGG cases presenting methylation-subtype pairs. (D) Fusions associated with young adult versus later-onset cancer cohorts. For each fusion event, a log10-transformed coefficient >0 represents a higher rate in young adult cases, while a log10-transformed coefficient <0 represents a higher rate in later-onset cases. Significant (FDR <0.05) and suggestive (FDR <0.15) fusions are labeled. See also Figure S4 and Table S3.

Using the PanCanAtlas prioritized gene-level methylations (Sanchez-Vega et al., 2018), we applied the multivariate model to identify events showing different rates in young adults cases (STAR Methods). We found six significant (FDR <0.05) and seven suggestive methylations affecting seven genes (Figure 4A; Table S3). Only three of the 13 identified events exhibited higher prevalences in young adult cancers: *LATS2* methylation was strongly associated with young adult gliomas (FDR = 7.92E-9), while methylations of *MGA* (FDR = 0.017) and *CDKN2A* (FDR = 0.085) were significantly and suggestively associated with young adult KIRP, respectively. Notably, *CDK2NA* methylation was also significantly associated with later-onset LIHC (FDR = 1.40E-5) and SKCM (FDR = 0.017), and suggestively associated with later-onset THCA (FDR = 0.057) and BRCA (FDR = 0.069) (Figure 4A). Methylations of *HES4* (FDR = 0.017) and *NOV* (FDR = 3.26E-3) were each significantly associated with later-onset gliomas, while *TCF7* methylation was suggestively associated (FDR = 0.132). Additionally, *TCF7* methylation (FDR = 0.102) and *MGA* methylation (FDR = 0.069) were each suggestively associated with later-onset BRCA, while a suggestive association was identified between *TLE3* methylation and later-onset LIHC (FDR = 0.079). The prevalence of *TCF7* methylation and *CDK2NA* methylation (except for *CDK2NA* in KIRP) were enriched in the later-onset cases across multiple cancer types (Figure 4A). In parallel, age-as-a-continuous-variable regression analysis re-discovered that *LATS2* and *CDK2NA* methylation rates significantly decreased with age in LGG (FDR = 4.68E-3) and HNSC (FDR = 0.046), respectively (Figure S4A).

Comparing methylation-subtype pairs within cancer types to their young adult versus later-onset status (STAR Methods), we identified five pairs showing significant and one showing suggestive age-related enrichments, none of which appeared in BRCA (Figures 4B and S4B). We found *LATS2*-methylated IDHmut-non-codel gliomas to be significantly enriched in young adults (FDR= 1.21E-13), while *LATS2*-methylated IDHmut-codel gliomas were suggestively enriched in later-onset cases (FDR = 0.100) (Figures 4C and S4C). *HES4*-methylated IDHwt (FDR = 9.58E-3) and *NOV*-methylated IDHwt (FDR = 0.010) gliomas were both significantly enriched in the later-onset cohort, along with *HES4*-methylated IDHmut-codel (FDR = 0.015) and *TCF7*-methylated IDHmut-codel (FDR = 0.016) gliomas (Figures 4C and S4C). These results demonstrate that age-related methylations in LGG are particularly subtype specific, while those in BRCA are not.

We also applied the multivariate model to identify fusion drivers characterized by PanCanAtlas (Sanchez-Vega et al., 2018), finding three fusions showing suggestive associations with onset age status (Figure 4D; Table S4). Suggestive associations were found between young adult THCA and gene fusions involving *RET* (FDR = 0.102) or *NTRK3* (FDR = 0.102), as well as between later-onset gliomas and *EGFR* fusions (FDR = 0.102) (Figure 4D). Fusion-subtype pairs identified enrichment of *EGFR*-fusion IDHwt gliomas in the later-onset cohort (STAR Methods; Figures S4D and S4E).

### Gene and pathway expression differences

**Figure 5.**
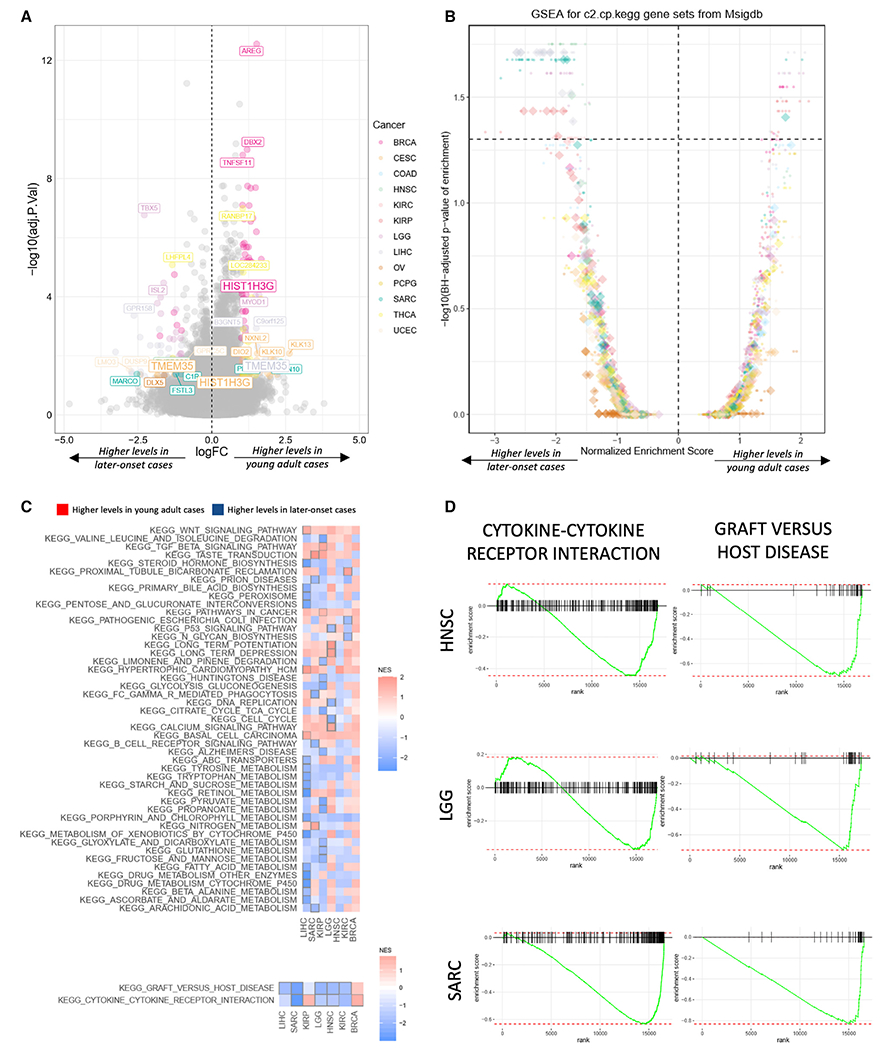
Differentially expressed genes and pathways between young adult and later-onset tumors (A) Differentially expressed genes between young adult and later-onset cancer cohorts. Positive and negative logFC values represent higher levels in young adult and later-onset cases, respectively. Color represents a gene differentially expressed in a specific cancer type; gray represents a gene not differentially expressed in any cancer. Differentially expressed genes with BH-corrected p values less than that of the top third gene are labeled. (B) Overview of pathway perturbed by differential expression in different cancers. Positive and negative normalized enrichment scores represent higher levels in young adult and later-onset cases, respectively. (C) The normalized enrichment scores of pathway-cancer associations. Red and blue indicate higher levels in young adult and later-onset cases, respectively. Significant associations are pinpointed by gray boxes. Cancers are ordered by the number of significantly perturbed pathways involved, and pathways are split based on the number of involved cancers. (D) Enrichment of two immune-related pathways (cytokine-cytokine receptor interaction and graft versus host disease) with consistently lower expressions across three cancer types (HNSC, LGG, and SARC). See also Figure S5.

To elucidate mRNA-level differences between young adult compared to later-onset cases, we identified differentially expressed genes using a multivariate linear regression model implemented in limma (Ritchie et al., 2015), adjusting for subtype, tumor stage, gender, and genetic principle components (STAR Methods). We identified 207 instances of differentially expressed genes (FDR <0.05) across nine of the 13 analyzed cancer types with sufficient data (Figure 5A). Overall, 114 genes showed higher expression, which were found primarily in BRCA, CESC, LIHC, and THCA, compared to 93 genes showing lower expression in young adult cases (Figure S5A). The majority of differentially expressed genes (193) were identified in five cancer types (BRCA, CESC, LGG, LIHC, and THCA) (Figure S5A), suggesting their age-related differences in gene expression.

Next, we investigated pathways enriched by differentially expressed genes between young adult and later-onset cases using gene set enrichment analysis (GSEA) (Subramanian et al., 2005). We identified 158 instances of significantly differentially expressed pathways (FDR <0.05), involving 86 unique pathways across seven cancer types. Among them, 124 differentially expressed pathways showed negative normalized enrichment scores, indicating lower pathway gene-expression levels in young adult cases (Figures 5B and S5B). Two immune-related pathways, graft versus host disease and cytokine-cytokine receptor interaction, showed consistently lower expressions across cancer types (except for BRCA) and were significant in SARC, LGG, and HNSC (Figures 5C, 5D, and S5C), suggesting lower immune involvements in these young adult cases.

It has been hypothesized that metabolic dysregulation (e.g., induced by obesity) may have driven the recent rise in young adult cancer (Berger, 2018). From the pathway enrichment analyses, we identified 26 instances of differentially expressed metabolic pathways (Figure 5B). Surprisingly, 25 of the associations showed negative normalized enrichment scores, suggesting lower transcription levels of genes in these pathways compared to later-onset cases. Four pathways showed lower expression in two cancer types, including the linoleic acid pathway in LIHC (FDR = 0.030) and HNSC (FDR = 0.031), amino sugar and nucleotide sugar metabolism in SARC (FDR = 0.021) and KIRP (FDR = 0.034), and butanoate metabolism as well as glycine, serine, and threonine metabolism in both LIHC (FDR = 0.019) and KIRP (FDR ≤ 0.034) (Figure S5D). Genes involved in the type I diabetes mellitus pathway also showed significantly lower expression across four cancers: SARC (FDR = 0.021), LGG (FDR = 0.024), HNSC (FDR = 0.018), and KIRC (FDR = 0.049) (Figure S5D). Overall, these results implicated lower expression of metabolic genes in this series of young adult cancers, and further investigation of metabolic wiring across different types of young adult cancers is required.

### Tumor immune microenvironment

**Figure 6.**
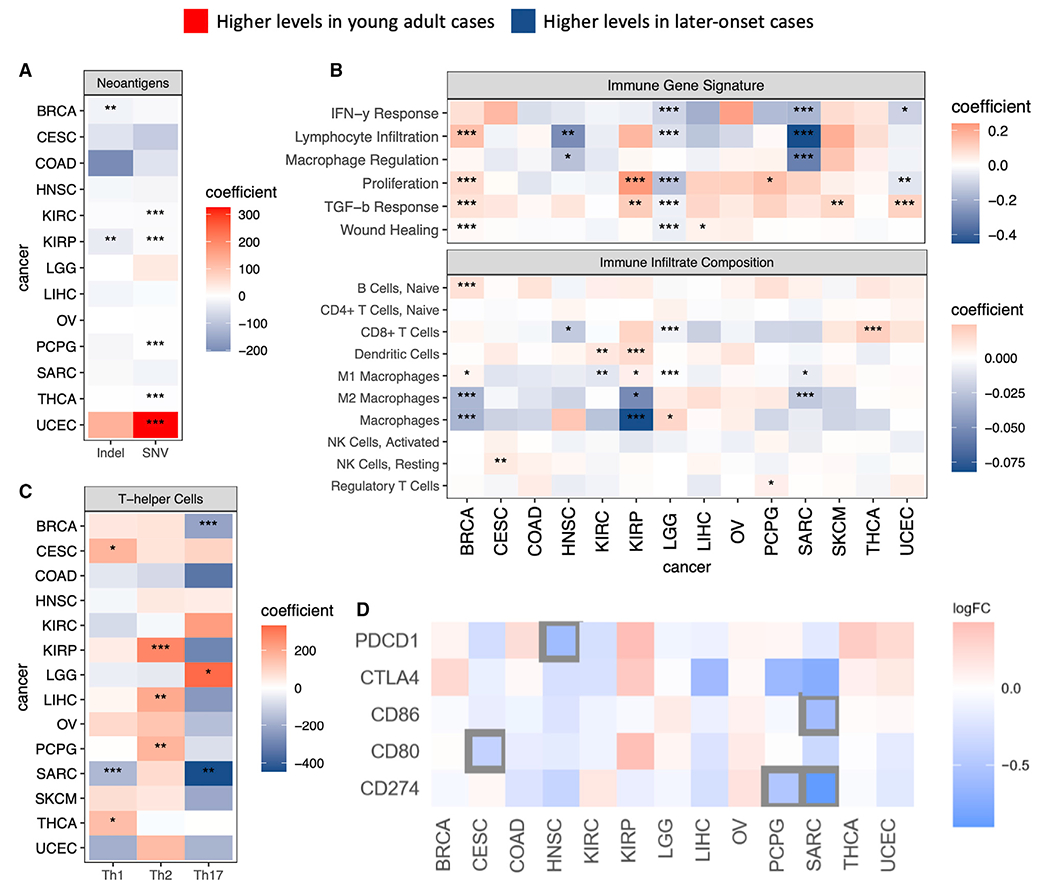
Tumor immune microenvironment differences between young adult and later-onset cases (A) Differences in neoantigen loads between young adult and later-onset cases. Red and blue indicate higher levels in young adult and later-onset cases, respectively. *FDR ≥0.10 and <0.15, **FDR ≥0.05 and <0.10, and ***FDR <0.05. (B) Differences in immune gene signatures and infiltrates between young adult and later-onset cases. Red and blue indicate higher levels in young adult and later-onset cases, respectively. *FDR ≥0.10 and <0.15, **FDR ≥0.05 and <0.10, and *** FDR <0.05. (C) Differences in Th cell levels between young adult and later-onset cases. Red and blue indicate higher levels in young adult versus later-onset cases, respectively. *FDR ≥0.10 and <0.15, **FDR ≥0.05 and <0.10, and ***FDR <0.05. (D) Positive logFC (red) indicates higher expression in young adult cases; negative logFC (blue) indicates down-expression in young adult cases. Genes with p < 0.05 are pinpointed by black boxes. See also Figure S6.

The tumor immune microenvironment (TIME) dictates patient prognosis and response to immunotherapy (Binnewies et al., 2018), but TIME in young adult cases remain poorly characterized. Tumor neoantigens can be susceptible to recognition by the adaptive immune system (Ward et al., 2016), and we utilized the multivariate model to identify potential differences in neoantigen loads between young adult and later-onset cases (STAR Methods). With few exceptions, higher SNV and indel neoantigen loads were associated with later-onset cancers (Figure 6A), concordant with their higher mutation rates (Figure S1A). However, higher SNV neoantigen load was associated with young adult UCEC (FDR = 2.76E-9, Figure 6A), possibly due to the prevalence of the POLE subtype in young adult UCEC and its high mutation rates (Figure 1C). Accordingly, *POLE* mutations were previously found to precipitate a dramatic rise in SNVs, but not indels (León-Castillo et al., 2020; Temko et al., 2018).

While old age is associated with diminished immune responses (Montecino-Rodriguez et al., 2013; Simon et al., 2015), its effects on the TIME remain to be elucidated. To this end, we examined the young adult versus later-onset differences in immune gene signatures, immune infiltrates, and Th and other immune cell fractions derived from gene-expression data (STAR Methods) (Thorsson et al., 2018). Across cancer types, we found, on average, lower interferon (IFN)-γ responses and lymphocyte infiltration in young adult cases, including significant associations in LGG and SARC. In contrast, greater transforming growth factor (TGF)-β response was associated with young adult cancer, showing significant associations in BRCA/UCEC (FDR <0.050) and suggestive in KIRP/SKCM (FDR <0.084). Intriguingly, compared to later-onset cases within their respective cancer cohorts, young adult BRCA exhibited more robust immune signatures, whereas young adult LGG showed diminished tumor immune response signatures, including a significant decrease in TGF-β response (Figure 6B). We next evaluated the cell-type compositions in TIME: the majority of young adult cancers exhibited higher naive B cell levels but reduced macrophages, including significant decreases in BRCA/KIRP total macrophages and BRCA/SARC M2 macrophages. We also noted a trend of increased dendritic cells among young adult cases, reaching significant/suggestive levels in both renal cancers (Figure 6B). Th cell analysis found elevated Th2 levels, connected to generally unfavorable outcomes across cancer types (Lee et al., 2019; Protti and De Monte, 2012), to be significantly associated with young adult KIRP (FDR = 0.035). Conversely, elevated Th1 and Th17 levels, both of which have been linked to improved cancer outcomes (Kim and Cantor, 2014; Maimela et al., 2019; Thorsson et al., 2018), were significantly associated with later-onset SARC (FDR = 1.52E-3) and BRCA (FDR = 0.025), respectively. Across cancer types, stronger Th2 and weaker Th17 responses were consistently salient features of the young adult cohort (Figure 6C). A multivariate regression analysis treating age as a continuous variable confirmed multiple key findings, including increasing SNV neoantigen loads, macrophages, and Th17 cells with age in UCEC, KIRP, and BRCA, respectively (Figures S6A–S6C). We also found significantly and suggestively higher leukocyte fractions in later-onset SARC (FDR = 6.82E-3) and young adult BRCA (FDR = 0.105), respectively (Figures S6D and S6E).

These results were corroborated by our expression analysis of immune markers using RNA sequencing (RNA-seq) data (Figure 6D), which found *PDCD1* (PD-1) expression, a co-inhibitory checkpoint protein expressed by activated CD8^+^ T cells (Simon and Labarriere, 2018), to be higher (p < 0.05) in later-onset HNSC, which also showed suggestively elevated CD8^+^ T cells (Figure 6B). Our immune marker analysis also found *CD274* (PD-L1) expression, strongly associated with tumor macrophages (Goldman et al., 2018), to be elevated in later-onset SARC (Figure 6D). For young adult cases, several immune checkpoint genes showed trends of elevated (albeit not significant) expression in KIRP (which had significantly higher dendritic cell fractions), suggesting these tumors may be suitable for treatments using immune checkpoint inhibitors.

We next sought to evaluate the potential therapeutic implications of the age-related differences in TIME. Utilizing a cohort of 1,525 patients of multiple solid tumor types who received immune checkpoint inhibitors (Samstein et al., 2019), we conducted a survival analysis adjusting for tumor mutational burden (TMB) within each cancer type (STAR Methods). We found a trend of young adult HNSC (p = 0.068) and BLCA (p = 0.086) presenting with worse post-immunotherapy survival (Figure S6F). The worse prognosis for young adult HNSC parallels the corresponding TCGA cases’ lower levels of lymphocyte infiltration and CD8^+^ (cytotoxic) T cells compared to later-onset HNSC. Additionally, we observed higher levels of naive CD4^+^ T cells—a negative prognostic factor in the TIME (Su et al., 2017)—among young adult HNSC (Figure S6B). These results implicate that the age-associated TIME differences we identified using multi-omic analyses may be correlated with different responses in age-stratified patient populations treated with immunotherapy and require further clinical validation.

### Clinically actionable events

Complementing features of TIME that can help determine immunotherapy response, the different genomic events observed in young adult cancer can guide other targeted treatments. We next identified biomarkers of clinical actionability by compiling data from three databases: CIViC (Griffith et al., 2017), CGI (Tamborero et al., 2018), and OncoKb (Chakravarty et al., 2017). We established an analysis workflow identifying genomic markers linked to both approved (level A) and experimental (level B) anti-cancer drugs that appear most frequently in young adult cancers, as well as whether a given patient’s cancer type is an approved indication for the drug (on versus off-label status). To arrive at a reasonable estimate of fractions of patients that may benefit from therapies, we focused on seven cancers (BRCA, CESC, COAD, HNSC, SKCM, THCA, and UCEC) that had ≥10 young adult and ≥10 later-onset cases with actionable mutations at the A or B evidence levels in this analysis (STAR Methods).

**Figure 7.**
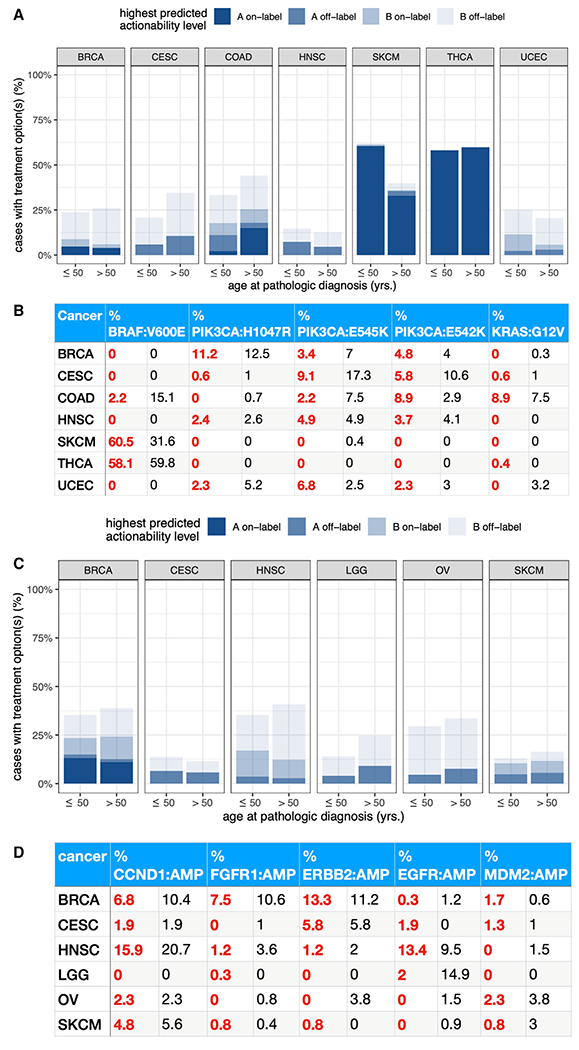
Clinical actionability in young adult versus later-onset tumors (A) Percentages of unique young adult and later-onset cases with somatic variants druggable at the A or B evidence levels, further subdivided by on versus off-label status. (B) Percentages of unique young adult and later-onset cases expressing each of the top five (ranked by frequency of appearance in the seven selected cancers) clinically druggable somatic variants. Young adult percentages are red; later-onset are black. After correcting for confounding factors, a single significant association (FDR <0.05) was found between *BRAF* V600E mutations and young adult SKCM. (C) Percentages of unique young adult and later-onset cases with copy-number amplifications druggable at the A or B evidence levels, further subdivided by on versus off-label status. (D) Percentages of unique young adult and later-onset cases expressing each of the top five (ranked by frequency of appearance in the six selected cancers) clinically druggable copy-number amplifications. Young adult percentages are red; later-onset are black. See also Figure S7.

*BRAF* V600E, a recurrent biomarker for approved drugs (level A) in multiple cancer types, shows diverging associations with young adult status. In COAD, 15.1% of later-onset cases, compared to only 2.2% of young adult cases, harbored mutations in *BRAF* V600E, which was druggable with two combination therapies (Panitumumab + Encorafenib and Encorafenib + Cetuximab); these fractions account for all COAD cases with level A on-label treatment options in both cohorts (Figures 7A, 7B, and S7A). In contrast, 60.5% of young adult SKCM harbor *BRAF* V600E (all young adult Level A on-label SKCM cases) compared to only 31.6% of later-onset cases (Figure 7B), potentially druggable with multiple A on-label therapeutics such as Vemurafenib, Trametinib, Dabrafenib + Trametinib, Cobimetinib + Vemurafenib. These BRAF associations were concordant with trends reported by previous studies in COAD (Chen et al., 2014) and SKCM (Bauer et al., 2011). We also observe clear differences at the A off-label level for multiple cancers types: 10.6% of later-onset versus 5.8% of young adult CESC were maximally treatable at this level, compared to 8.9% of young adult versus 2.9% of later-onset COAD (Figures 7A and S7A). Across cancer types, *PIK3CA* H1047 mutations show comparable frequencies between young adult and later-onset cases, while *PIK3CA* E545K and E542K hotspot mutations display age dependence, most notably in BRCA, CESC, COAD, and UCEC. In contrast, frequencies of druggable *KRAS* hotspot mutations do not appear to be age-related in any of the seven cancer types (Figures 7B and S7B). We note these descriptive frequencies in young adult versus later-onset groups do not indicate age associations that are independent of other clinical variables. Correcting for confounding factors, we found *BRAF* V600E mutations to be significantly associated with young adult SKCM (Figure S7C, FDR = 4.60E-5). We also found an association between *BRAF* V600E mutations and later-onset COAD (p = 3.13E-3), as well as associations between *PIK3CA* E545K mutations and later-onset BRCA, CESC, and COAD (P ≤ 0.0367); however, they did not reach the significance level for this study (Figure S7C).

We next examined the clinically actionable copy-number amplifications, where six cancers (BRCA, CESC, HNSC, LGG, OV, and SKCM) satisfied the minimum required sample size for analyses. In BRCA, we found a slightly higher rate of the A on-label *ERBB2* copy-number amplification in young adult cases (13.3%) compared to later-onset cases (11.2%), representing all BRCA cases treatable with A on-label drugs (Figures 7C, 7D, and S7D). All other cancer types only had A off-label copy-number amplifications, and later-onset cases generally showed similar or slightly higher frequencies of actionable events. In LGG, A off-label was the highest predicted actionability level for 27.5% of later-onset versus 13.5% of young adult cases (Figures 7C and S7D). A large proportion of the discrepancy can be explained by 7.8% of later-onset cases compared to 2.6% of young adult LGG cases presenting *CDK4* amplification (Figure S7E), which corresponded to the A off-label combination therapy Abemaciclib + Palbociclib. In the multivariate analysis correcting for covariates (Figure 3B), *ERBB2* copy-number amplification was not significantly associated with age in BRCA (p = 0.35), whereas CDK4 amplification was associated with later-onset LGG (p = 0.010), validating recently published findings in a Japanese glioblastoma cohort (Fukai et al., 2020). Overall, while our results highlight age-related molecular drivers across cancer types, further studies are required to establish the mechanistical underpinning and clinical implications of their age associations in cancer.

## DISCUSSION

We present here a comprehensive investigation of young adult cancer’s genomic and molecular profiles, uncovering molecular features distinguishing cancer of this understudied population suffering from increased incidence in the US and worldwide (Gupta et al., 2020; Sung et al., 2019).

While accumulated mutations throughout an individual’s lifetime are known to drive tumorigenesis (Stratton et al., 2009; Tomasetti et al., 2013; Vogelstein et al., 2013), young adult cancers may be precipitated by specific, aggressive genomic drivers. For example, while *IDH1/2* mutations are known to be associated with young adult gliomas (Haase et al., 2018; Yan et al., 2009), our paired mutation-subtype analysis provides improved resolution, showing IDHmut-non-codel glioma enrichment in young adults whereas IDHmut-codel gliomas are enriched in later-onset cases (Figure 1B). Building on this distinction, we also found mutations in *TP53* and *ATRX* to be associated specifically with IDHmut-non-codel subtype gliomas (Brat et al., 2015; Cheung et al., 2012) (Figures 2A and 2C). Additionally, we confirmed the association between *PTEN* mutations and young adult COAD found in previous reports (Ballester et al., 2016; Berg et al., 2010). Interestingly, we also identified mutations in both *ATRX* and *PTEN* associated with young adult UCEC (Figure 2A), suggesting a pan-cancer relationship between these gene-level mutations and young adult cancer.

Copy-number variations dominate the functional genomic drivers in multiple cancer types and may explain young adult tumors lacking driver mutations. Multiple copy-number amplifications are enriched in young adult cases, including *RPTOR* in BRCA, *ARRDC1/KRAS* in LGG, and *MYC* in OV. *RPTOR* amplification was specifically enriched in basal and luminal A BRCA in young adults (Figure 2D). Recent studies have linked *RPTOR* activity with treatment implications in triple-negative and luminal-A breast cancer models (Bostner et al., 2018; You et al., 2018). More, the druggable *ERBB2* amplification occurred in a quarter of young adult breast cancer cases. In young adult gliomas, we identified enrichment of *KRAS* and *CCND2* amplifications with potential targeted treatment options, whereas later-onset LGG showed higher rates of actionable *EGFR* amplification (Figure 7D).

While age strongly affects innate and adaptive immunity (Montecino-Rodriguez et al., 2013; Simon et al., 2015), its effects on the TIME remain unclear. Cancer types showing the greatest differences in young adults included BRCA, KIRC/KIRP, LGG, and SARC. Young adult status is associated with multiple prognostically unfavorable immune gene signatures in BRCA, including elevated TGF-β response and wound healing signatures but reduced Th17 levels (Kim and Cantor, 2014; Massagué, 2008; Thorsson et al., 2018) Figure 6B). Young adult sarcomas present diminished lymphocyte infiltration, IFN-γ response, M1 macrophages, and Th1 response (Figures 6B and 6C): together, these features would predict reduced survival time and poor treatment response (Brown et al., 2017; Lugade et al., 2008; Maimela et al., 2019; Thorsson et al., 2018). In KIRP, young adult cases present elevated TGF-β response and proliferation; however, they are also associated with a favorable increase in M1 macrophages, a feature shared with young adult BRCA (Brown et al., 2017; Massagué, 2008; Thorsson et al., 2018) (Figure 6B). While KIRC/KIRP and LGG show more prognostically mixed TIME features between cohorts, these findings would predict poorer overall survival in young adult BRCA and SARC (not considering other molecular or systematic differences). Intriguingly, an epidemiological review has shown that breast cancer and soft-tissue sarcomas are among the five cancer types that have worse 5-year survivals in young adults and adolescents (defined as 15–39 years at diagnosis) than in either older adults or children (Bleyer et al., 2008), corroborating our immune prognostic findings. This same review also found colorectal cancer to be among these cancer types showing worse prognosis for young adults (Bleyer et al., 2008). In our immunotherapy survival analysis, young adult BRCA and COAD also showed trends of worse survival (Figure S6F). Although the significance only borderlines the threshold of p < 0.05, the corroborating findings of TIME differences using multi-omic analyses suggest age may serve as additional factor that can affect immune response and should be considered, especially when comprehensive immune-profiling data are not readily available. Overall, TIME differences in young versus older adults vary across tissues, which may be a result of the complex interplays between accumulated neoantigens and age-related immune changes that require further investigation. These differences can determine patient prognosis and suitability of immunotherapy.

Overall, our comprehensive assessment revealed the molecular etiologies of young adult tumors across multiple cancer types. The findings highlighted key genomic and microenvironment alterations that may be targeted by kinase inhibitors and immunotherapies, presenting possible treatment options for young adult cancer patients.

### Limitations of the study

While we identified multiple types of aberrations associated with young adult cancer in TCGA, studies using specifically designed, prospective tumor cohort collections are required to interrogate whether these molecular events are also the underlying drivers in the recently arising cases of young adult cancer. More, cohorts with detailed documentation on environmental factors are required to reveal how changes in diet or lifestyle interacted with the molecular drivers. For example, at the mRNA level, we found a general trend of lower metabolic gene expressions in young adult cancer. While obesity is a risk factor for multiple cancer types and some evidence has paired the obesity pandemic in the US to its rising rates in young adult cancer (Berger, 2018), our gene-expression analyses revealed these tumors, except for BRCA, generally showed lower metabolic gene expression after correcting for tumor stage and subtypes (Figures 5C and S5D). The metabolic regulations in young adult cancer remain to be further investigated. Last, while we identified age-related differences in clinically actionable drivers and the tumor immune microenvironment, their possible implication on treatment also warrants further mechanistic and clinical studies.

## STAR★METHODS

### RESOURCE AVAILABILITY

#### Lead contact

Further information and requests for resources and data should be directed to and will be fulfilled by the lead contact, Dr. Kuan-lin Huang (kuan-lin.huang@mssm.edu).

#### Materials availability

This study did not generate new unique reagents.

#### Data and code availability

- This paper analyzes existing, publicly available data. DOIs or links for the datasets are listed in the key resources table. The TCGA genomic data used herein are generated from the TCGA PanCanAtlas project.
- All original code has been deposited at Zenodo and is publicly available as of the date of publication. DOIs are listed in the key resources table.
- Any additional information required to reanalyze the data reported in this paper is available from the lead contact upon request.

### METHOD DETAILS

#### Cohort and data description

Based on 10,956 unique TCGA cases, we only included samples that had available gender, age at initial pathologic diagnosis, and principal component data as calculated by the PanCanAtlas Germline project (Huang et al., 2018; Oak et al., 2020). In the 7 cancer types with available subtype information, we further filtered for cases with defined subtypes. We retained 8,943 unique cases across the 33 TCGA cancer types. Only cancer types with ≥ 40 cases in both the young adult and later-onset cohorts were retained for subsequent analyses, resulting in 14 cancer types and a total of 5,365 unique cases for downstream analyses. In the non-hypermutator somatic mutation analysis, the matching samples from the list of 342 hypermutated samples as defined by the TCGA PanCanAtlas project (Bailey et al., 2018) were removed before the multivariate regression analysis was conducted.

#### Somatic driver mutations

Somatic mutations of 10,244 cases were obtained from the Multi-Center Mutation Calling in Multiple Cancers (MC3) dataset (Ellrott et al., 2018). We only considered the nonsynonymous mutations in 299 cancer driver genes as defined by the PanCanAtlas driver project (Bailey et al., 2018), including missense, non-sense, frameshifting, in-frame shifting, or splice-site altering single-nucleotide changes or indels. Mutations predicted as functional impact by any of the algorithms described in Bailey et al. were considered as somatic driver mutations. Truncations in the 299 driver genes were also considered as drivers. We collected 35,815 likely somatic driver mutations for analyses.

#### DNA methylation

We obtained 16 prioritized gene-level driver methylations of 9,125 TCGA cases as defined by the PanCanAtlas project. Potential methylations were identified using the bioinformatics tool RESET, then retained only if they overlapped promoters also identified by FANTOM5 project. Further filtering excluded events with an FDR ≥ 10% or a RESET score ≤ 1, in addition to the manual removal of tissue-associated cases (Sanchez-Vega et al., 2018).

#### Copy number variations

We obtained 102 prioritized driver deletions and amplifications of 9,125 TCGA cases as defined by the PanCanAtlas project. Potential CNVs were identified through integration of GISTIC 2.0, which produced a list of statistically recurrent copy-number altered regions of interest (CL = 0.95). To filter for functional gene-level CNVs inside these regions of interest, only CNVs directly relevant to the function of a given gene (either oncogene or tumor suppressor) or designated as *oncogenic, likely oncogenic*, or *predicted oncogenic* in OncoKb were retained (Sanchez-Vega et al., 2018). Copy number values for each genomic segment were determined by applying ABSOLUTE to somatic DNA copy number data from 10,552 TCGA samples, and totals were determined by summing the number of detected segments within a given sample (Taylor et al., 2018).

#### Fusion events

We obtained 124 prioritized driver fusion events of 9,125 TCGA cases as defined by the PanCanAtlas project. Potential fusion events were called from TCGA RNA-Seq data (mapped to the human genome) using STAR-Fusion, in conjunction with EricScript and BREAKFAST algorithms. These fusion events were further filtered by their OncoKb labels: only those designated as *oncogenic, likely oncogenic*, or *predicted oncogenic* were retained (Sanchez-Vega et al., 2018).

#### Expression data

The batch-normalized mRNA gene expression data of TCGA samples were obtained from the PanCanAtlas consortium. For patients with data from multiple samples, we averaged the expression values. We next removed patients lacking any information of the following information: age, gender, PC1, PC2. The expression value of each gene is log2(expression+0.05) transformed. Only cancers with at least 40 patients in either young adult or later-onset cohorts were retained, resulting in 5,009 patients across 13 cancer types and 20,531 analyzed genes.

#### Genomic and immune signatures

We integrated genomic and immune signatures of 11,080 TCGA individuals from Thorsson et al., collected into a pan-cancer TIME immune feature matrix (Thorsson et al., 2018).

#### The ICGC validation cohort

To validate the age-related associations, we applied similar analyses on the ICGC somatic mutation and CNV data. The ICGC cohort, after filtering out all TCGA samples and samples missing necessary variables (e.g., age at pathologic diagnosis), contained 1,820 unique samples. We then filtered to cancers with corresponding types in the 14 retained TCGA cancers. LICA, LINC, and LIRI were collapsed into one acronym to match TCGA’s liver cancer designation: LIHC. Additionally, MELA cases were relabeled as SKCM to match TCGA’s melanoma designation. RECA covers 2 of the 14 retained TCGA cancer types: KIRC and KIRP. Ultimately, we ended up with 6 consolidated ICGC cancer types and a total of 642 unique samples to validate against our TCGA results. In the ICGC driver catalog, we removed any genes containing the following strings: ‘lncrna’, ‘enhancers’, and ‘telomere’. The ICGC multivariate regression model adjusted for these covariates: subtype, gender, and country (of the study).

### QUANTIFICATION AND STATISTICAL ANALYSIS

#### Multivariate regression model

Considering age effects on molecular profiles may be confounded by other factors, we evaluated standard ways to address such confounders, including multivariate regression and propensity score matching. Whether propensity score matching yields more robust findings compared to multivariate regression remains debated, and in practice the results are often similar (Biondi-Zoccai et al., 2011). A Monte Carlo simulation study showed that, when the number of events per confounder is greater than 8, logistic regression should be the technique of choice: its median percentage of bias reduces while the number of events increased above 8, in contrast to the propensity score approach, where the bias remains steady and is therefore preferable for smaller sample sizes (Cepeda et al., 2003). In the analyzed TCGA cancer cohorts, we have a median of 80 young adults in each cancer type and 4 covariates, equivalent to 20 events per confounder. Ergo, we chose multivariate regression models for subsequent analyses.

We considered age at initial pathologic diagnosis – our independent variable (x_i_) – as both binary and continuous across all analyses (except gene expression, where age was considered only as binary). In our age-as-binary analyses, subjects who were over 50 years old at initial pathologic diagnosis were classified as later-onset cases, while subjects 50 years or younger were classified as young adult cases. The dependent variable (y_i_) was the analysis-dependent feature of interest (e.g., somatic mutation status, methylation status, score for an immune feature, etc.). For all analyses except the immune feature analysis, the dependent variable was binary (i.e., the genetic event did or did not occur). For these analyses the family of our generalized linear model (GLM) function was set to “binomial,” and the test parameter of the following ANOVA function was set to “Chisq.” For the immune features analysis, the dependent variable was continuous. Ergo, for this analysis the family of our GLM was set to “gaussian,” and the test parameter of the following ANOVA function was set to “F.” Our covariates were subtype (in the 7 cancer types where it is available), gender (when cancer type was not female-specific), and the PC1 and PC2 that accounted for 80.8% of the variation across the first 20 principal components (Huang et al., 2018; Oak et al., 2020). P values were adjusted to FDR using the standard Benjamini-Hochberg (BH) procedure. In this study, significance was defined as an FDR < 0.05.

To adjust for large-magnitude coefficients obtained during our CNV, methylation, and fusion age-as-binary analyses (for the purpose of plotting), we first made all coefficients positive, then applied a log10 transformation. Positive log10-transformed coefficients that were originally negative were multiplied by −1, and negative log10-transformed coefficients that were originally positive were also multiplied by −1. In this way, we were able to scale down large magnitude coefficients while preserving their original direction. This same transformation was also applied to the coefficients obtained during the somatic mutation and CNV ICGC validations.

#### Aneuploidy, copy number segment, and leukocyte fraction analyses

We used Mann-Whitney U tests to determine significant and suggestive differences in summed ABSOLUTE-determined copy number segments (Taylor et al., 2018), aneuploidy scores, and leukocyte fractions (Thorsson et al., 2018) between young adult and later-onset cases across the 14 cancer types. We utilized base R’s wilcox.test() function to obtain p values, which were then adjusted to FDR using the BH procedure.

#### Enrichment of event-subtype pairs

We first used Fisher’s exact tests to identify significant and suggestive differences in subtype proportions between young adult and later-onset cases within cancer types, adjusting p values to FDR using the BH procedure. We then used Fisher’s exact tests to identify significant and suggestive differences in rates of event-subtype pairs between young adult and later-onset cases. This was done within cancer types, and the events tested were those found to be significant or suggestive by the GLM. Additionally, these event-subtype tests (part of the somatic mutation, methylation/fusion, and CNV analyses) were done only for cancer types in which significant or suggestive differences in subtype proportions had been found between young adult and later-onset cases. P values were again adjusted to FDR using the BH procedure.

#### Differentially expressed genes and pathways

Within each cancer type, we applied a multivariate linear regression model to assess the significance P value and differential expression of each gene between young adult and later onset cohorts adjusting any available covariant (PC1, PC2, subtype, tumor stage, gender), implemented by the lmFit function of R package limma (Ritchie et al., 2015). Only genes with less than 30% of zero proportion in a specific cancer type were assessed. Genes with BH adjusted P value < 0.05 and absolute values of coefficient > 1 were defined as differentially expressed genes.

We then used Gene Set Enrichment Analysis (GSEA) to identify KEGG pathways affected by the differential expression of genes in each cancer (Subramanian et al., 2005). First, genes were ranked by their expression change between young adult and later-onset cases. Next, the ranked gene lists were subjected to GSEA analysis as implemented by the R package fgsea. Pathways with BH-corrected p values < 0.05 were regarded as significant.

#### Immunotherapy cohort survival analysis

The association between age of cancer onset and overall survival after immune checkpoint inhibitor therapy was assessed using a genomic cohort of 1,525 patients with advanced solid tumors at the Memorial Sloan Kettering Cancer Center (Samstein et al., 2019). Age of cancer onset was dichotomized into ≤ 50 or > 50 years. Cox proportional hazards models adjusting for tumor mutation burden (TMB) were used to test the association of age of cancer onset and overall survival. The analysis was stratified by cancer histology.

#### Clinical actionability analyses

We mapped our biomarkers of interest (somatic variant and amplification) to 3 oncology knowledge bases: CIViC (Griffith et al., 2017), CGI (Tamborero et al., 2018), and OncoKb (Chakravarty et al., 2017). We made sure to exclude biomarkers not linked to either approved (Level A) or experimental (Level B) anti-cancer drugs, since we are specifically interested in clinical actionability. Additionally, for both analyses, only cancer types with ≥ 10 unique young adult and ≥ 10 unique later-onset cases with biomarker(s) druggable at the A or B evidence levels were retained for subsequent analyses. We manually annotated both patient cancer types and CGI + OncoKb cancer types (CIViC was pre-annotated) with DOIDs from the disease ontology database to determine on versus off-label status.

## Supplementary Material

1

## Figures and Tables

**Table:** KEY RESOURCES TABLE

Reagent or resource	Source	Identifier
**Deposited data**		
MC3 somatic mutations	Ellrott et al., 2018	https://doi.org/10.1016/j.cels.2018.03.002
PanCanAtlas-defined driver genes	Bailey et al., 2018	https://doi.org/10.1016/j.cell.2018.02.060
PanCanAtlas-prioritized gene-level methylations	Sanchez-Vega et al., 2018	https://doi.org/10.1016/j.cell.2018.03.035
PanCanAtlas-prioritized copy number variations	Sanchez-Vega et al., 2018	https://doi.org/10.1016/j.cell.2018.03.035
Summed ABSOLUTE-determined copy number segments per sample	Taylor et al., 2018	https://doi.org/10.1016/j.ccell.2018.03.007
PanCanAtlas-prioritized fusion events	Sanchez-Vega et al., 2018	https://doi.org/10.1016/j.cell.2018.03.035
Batch-normalized mRNA gene expression data	PanCanAtlas	https://gdc.cancer.gov/about-data/publications/pancanatlas
Genomic and immune signature data	Thorsson et al., 2018	https://doi.org/10.1016/j.immuni.2018.03.023
Survival and associated genomic data	Samstein et al., 2019	https://doi.org/10.1038/s41588-018-0312-8
Clinically actionable CIViC biomarkers	Griffith et al., 2017	https://doi.org/10.1038/ng.3774
Clinically actionable CGI biomarkers	Tamborero et al., 2018	https://doi.org/10.1186/s13073-018-0531-8
Clinically actionable OncoKb biomarkers	Chakravarty et al., 2017	https://ascopubs.org/doi/10.1200/PO.17.00011
ICGC somatic mutation and copy number variation data	Campbell et al., 2020	https://doi.org/10.1038/s41586-020-1969-6
**Software and algorithms**		
R version 4.0.2	The R Foundation	https://www.r-project.org/foundation/
Gene Set Enrichment Analysis	Subramanian et al., 2005	https://doi.org/10.1073/pnas.0506580102
limma	Ritchie et al., 2015	https://doi.org/10.1093/nar/gkv007
Venn diagram tool	Van de Peer Lab	http://bioinformatics.psb.ugent.be/webtools/Venn/
Numbers version 10.0	Apple Inc.	https://www.apple.com/numbers/
Multivariate regression model	This paper; Zenodo	Zenodo: https://doi.org/10.5281/zenodo.5576639
Code for data cleaning, analysis, and plotting	This paper; Zenodo	Zenodo: https://doi.org/10.5281/zenodo.5576639

## Highlights

- Gene-level mutations, CNVs, methylations, and fusions associated with young adult cases
- Different rates of mutation/CNV-subtype pairs in young adult versus later-onset cases
- Pan-cancer approach identifies consistent age-related tumor immune response
- Young adult cases show different frequencies of actionable genomic drivers
