# Supplementary material for: Genomic and molecular features distinguish young adult cancer from later-onset cancer
Source: Cell Rep. Author manuscript; Available in PMC 2021 Nov 30. (PMC8631509; doi:10.1016/j.celrep.2021.110005)
Supplement: 1 [file NIHMS1757908-supplement-1.pdf]

Cell Reports, Volume *37*

Supplemental information

Genomic and molecular features distinguish young adult cancer from later-onset cancer

William Lee, Zishan Wang, Miriam Saffern, Tomi Jun, and Kuan-lin Huang

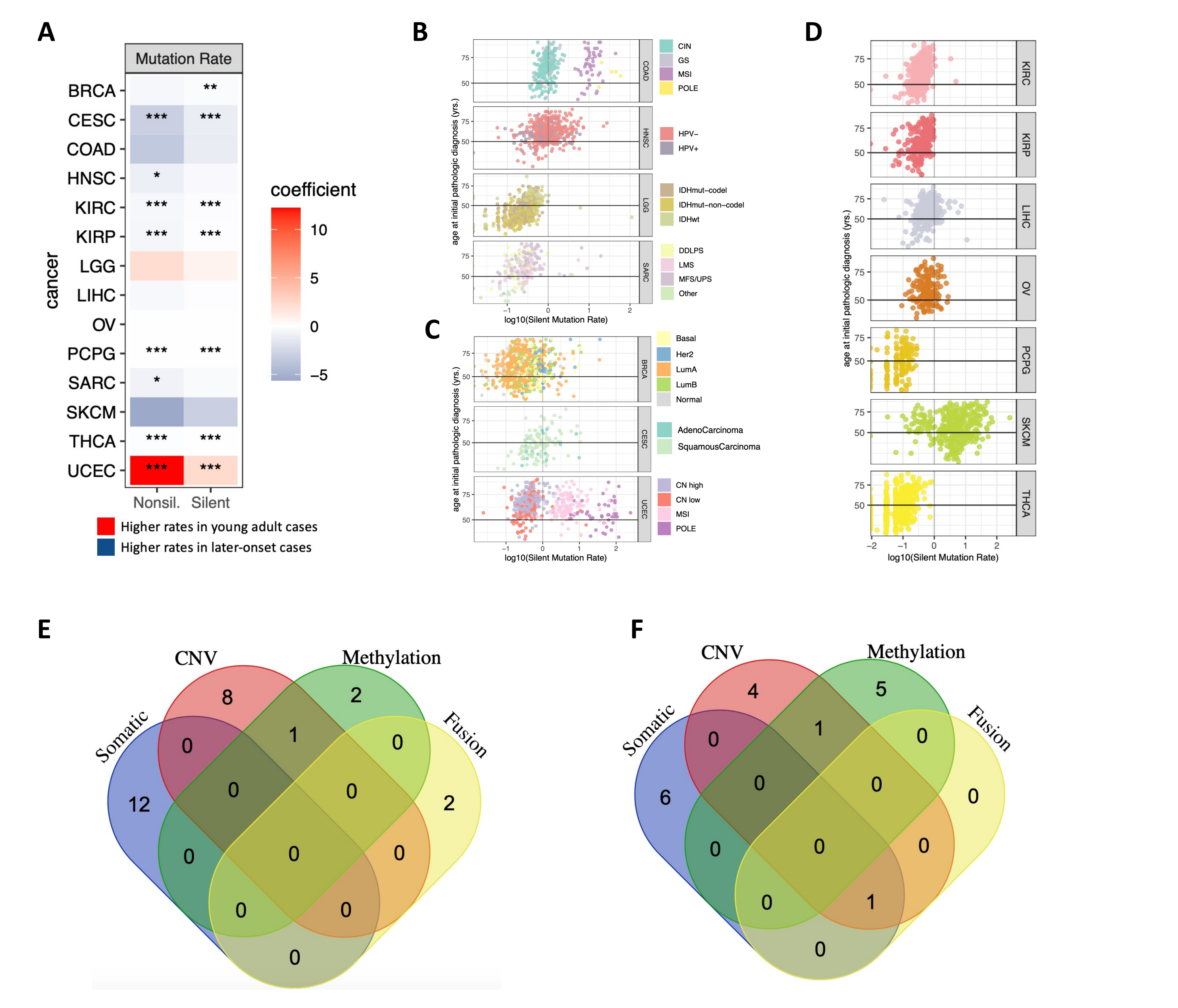

Figure S1. Silent and nonsilent mutation rates between young adult and later-onset tumors. Related to Figure 1 (A) Differences in silent and nonsilent mutation rates between young adult and later-onset cases. Red and blue indicate higher rates in young adult versus later-onset cases, respectively. * signifies an FDR ≥ 0.10 and < 0.15, ** signifies an FDR ≥ 0.05 and < 0.10, and *** signifies an FDR < 0.05. (B) Log10-transformed silent mutation rates for unique individuals in each non-sex-specific cancer type are colored by subtype. (C) Log10-transformed silent mutation rates for unique individuals in each sex-specific cancer type are colored by subtype. (D) Log10-transformed silent mutation rates for unique individuals in cancer types without subtype information are distinguished by PanCanAtlas colors. (E) Venn diagram showing number of overlapping genes between the somatic mutation, CNV, methylation, and fusion analyses among young adult cases. (F) Venn diagram showing number of overlapping genes between the somatic mutation, CNV, methylation, and fusion analyses among later-onset cases.

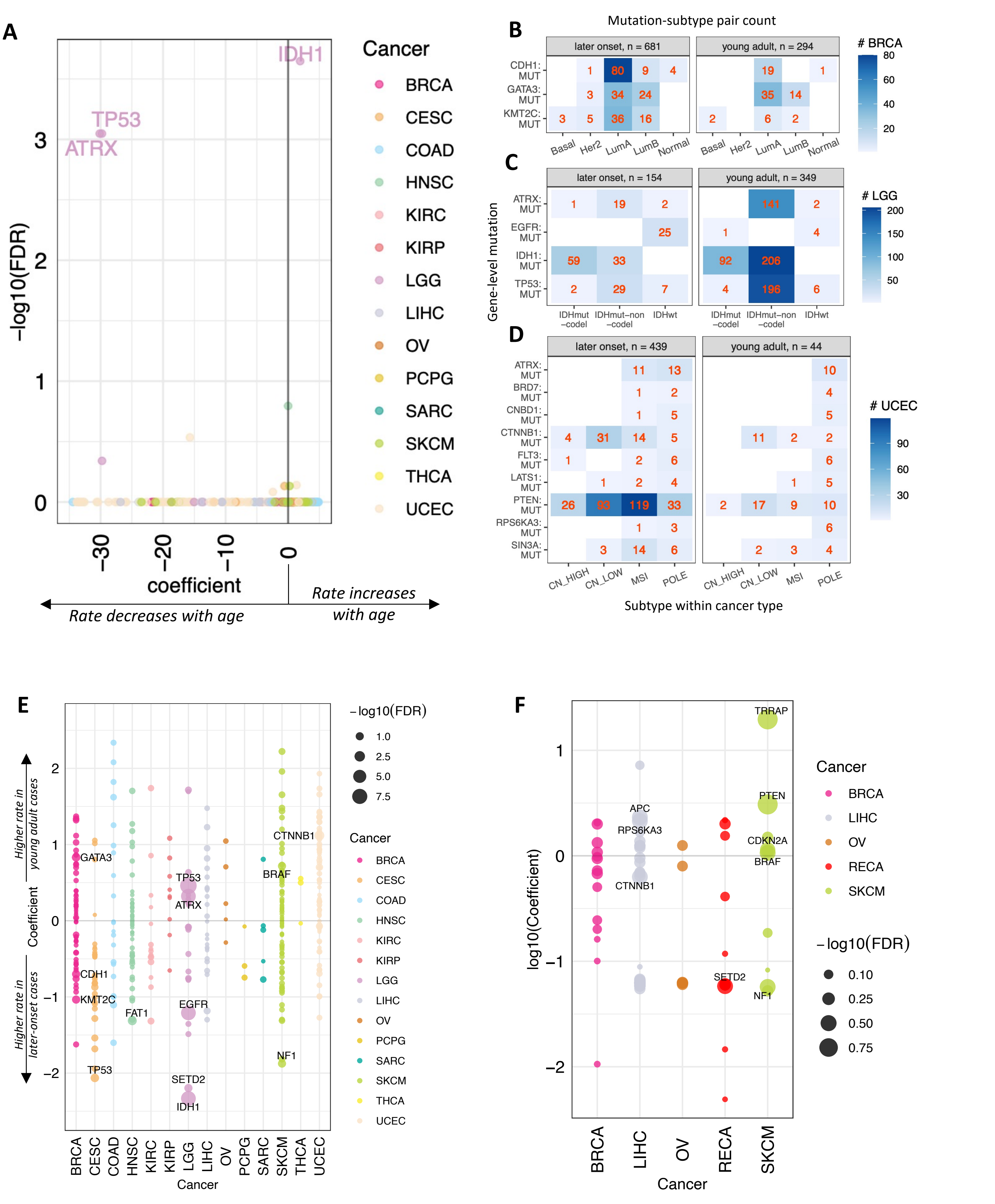

Figure S2. Additional investigation and validation of somatic mutations in young adult versus later-onset tumors. Related to Figure 2 (A) Somatic mutations with rates that increase or decrease with age. For each gene-level mutation, a coefficient > 0 indicates increasing rate with age, while a coefficient < 0 indicates decreasing rate with age. Significant mutations (FDR < 0.05) are labelled. (B) Counts of young adult vs. later-onset BRCA cases presenting mutation-subtype pairs. (C) Counts of young adult vs. later-onset LGG cases presenting mutation-subtype pairs. (D) Counts of young adult vs. later-onset UCEC cases presenting mutation-subtype pairs. (E) Non-hypermutator analysis. For each gene-level mutation, a coefficient > 0 indicates increasing rate with age, while a coefficient < 0 indicates decreasing rate with age. Significant mutations (FDR < 0.05) are labelled. (F) ICGC somatic mutation validation. For each gene-level mutation, a coefficient > 0 indicates increasing rate with age, while a coefficient < 0 indicates decreasing rate with age. Mutations with p-values < 0.05 are labelled.

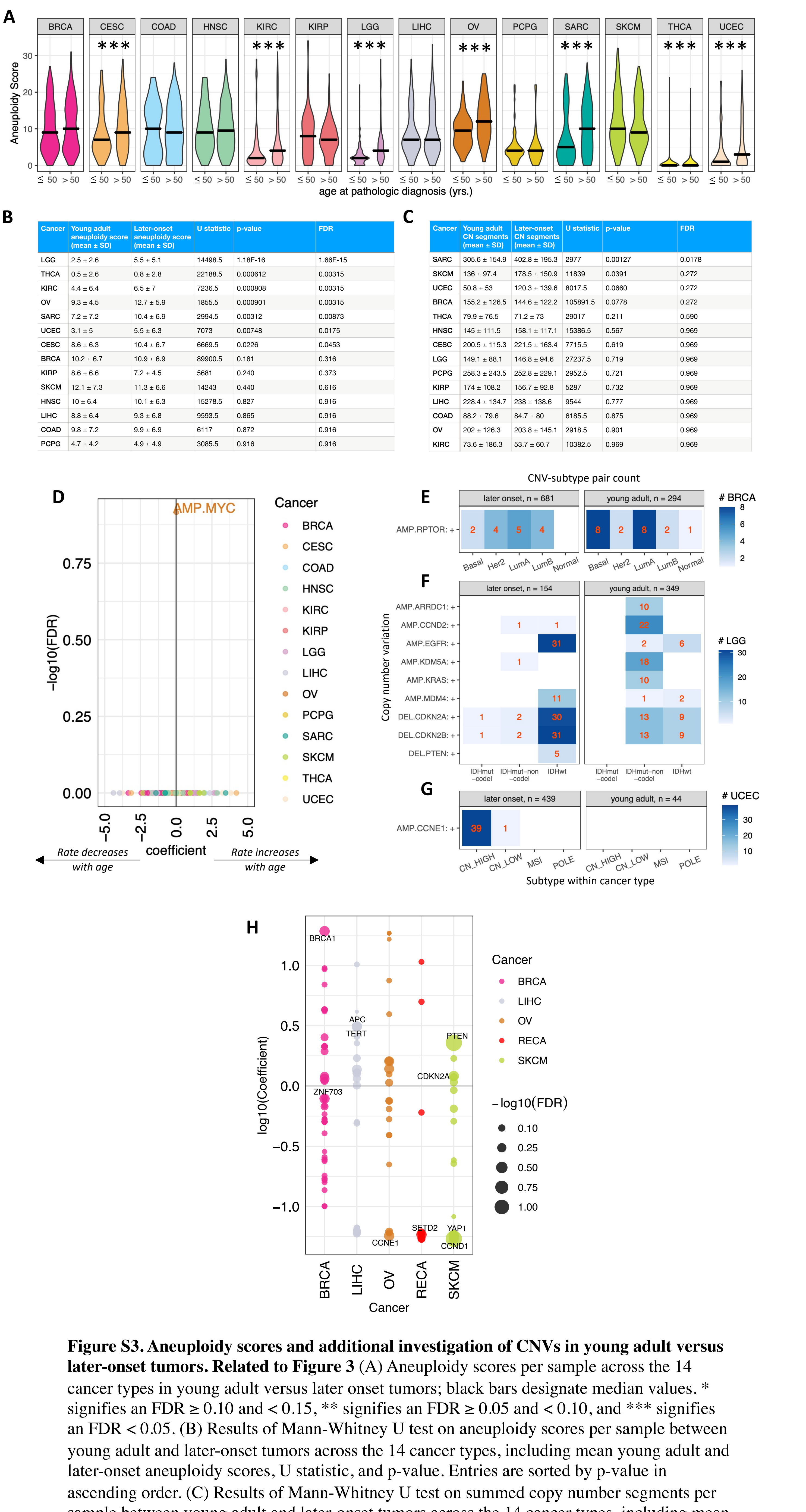

Figure S3. Aneuploidy scores and additional investigation of CNVs in young adult versus later-onset tumors. Related to Figure 3 (A) Aneuploidy scores per sample across the 14 cancer types in young adult versus later onset tumors; black bars designate median values. * signifies an FDR ≥ 0.10 and < 0.15, ** signifies an FDR ≥ 0.05 and < 0.10, and *** signifies an FDR < 0.05. (B) Results of Mann-Whitney U test on aneuploidy scores per sample between young adult and later-onset tumors across the 14 cancer types, including mean young adult and later-onset aneuploidy scores, U statistic, and p-value. Entries are sorted by p-value in ascending order. (C) Results of Mann-Whitney U test on summed copy number segments per sample between young adult and later-onset tumors across the 14 cancer types, including mean young adult and later-onset CN segments, U statistic, and p-value. Entries are sorted by p-value in ascending order. (D) CNVs with rates that increase or decrease with age. For each gene-level CNV, a coefficient > 0 indicates increasing rate with age, while a coefficient < 0 indicates decreasing rate with age. Significant (FDR < 0.05) and suggestive (FDR < 0.15) CNVs are labelled. (E) Counts of young adult vs. later-onset BRCA cases presenting CNV-subtype pairs. (F) Counts of young adult vs. later-onset LGG cases presenting CNV-subtype pairs. (G) Counts of young adult vs. later-onset UCEC cases presenting CNV-subtype pairs. (H) ICGC CNV validation. For each gene-level CNV, a coefficient > 0 indicates increasing rate with age, while a coefficient < 0 indicates decreasing rate with age. CNVs with p-values < 0.05 are labelled.

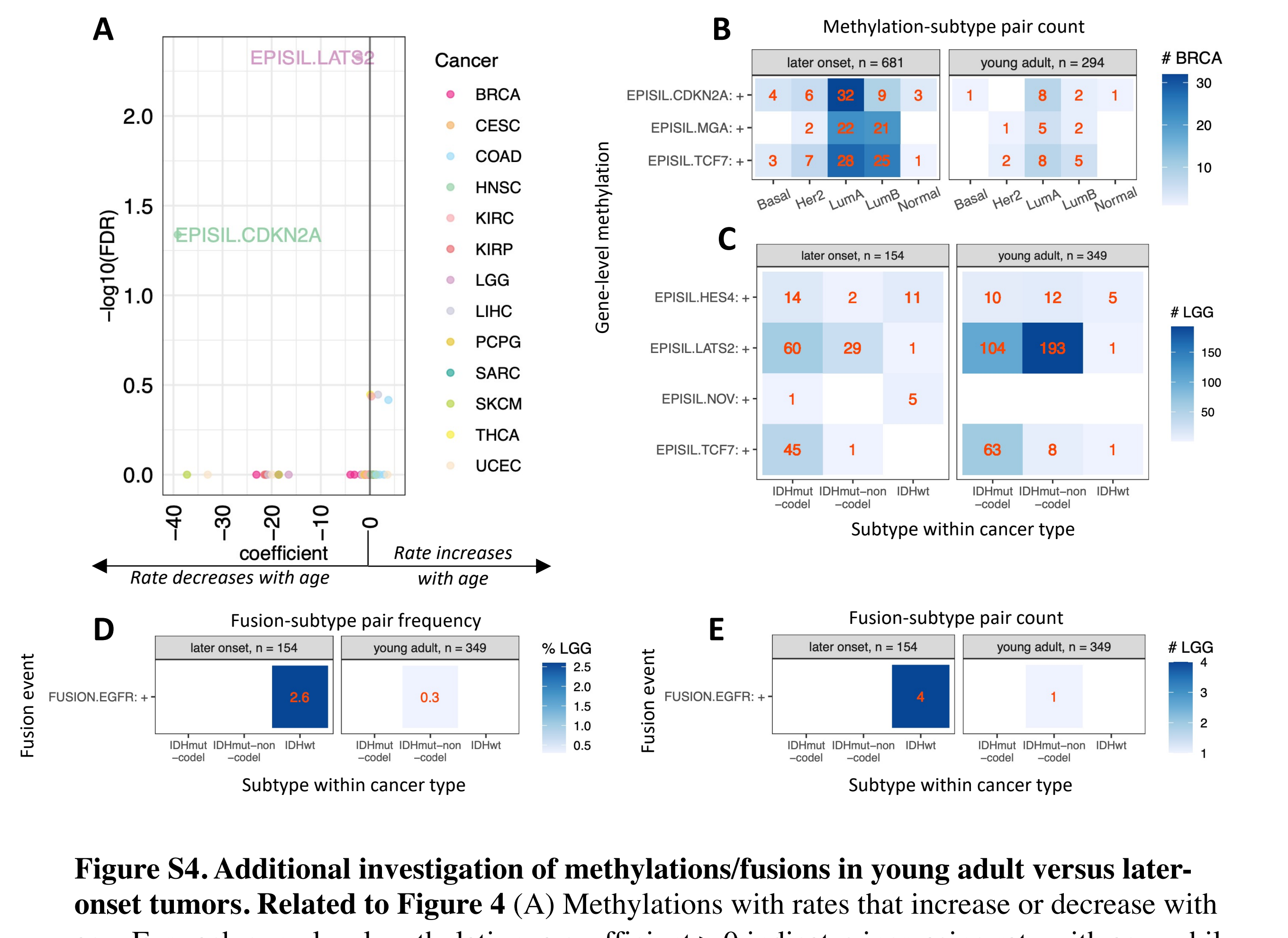

Figure S4. Additional investigation of methylations/fusions in young adult versus later-onset tumors. Related to Figure 4 (A) Methylations with rates that increase or decrease with age. For each gene-level methylation, a coefficient > 0 indicates increasing rate with age, while a coefficient < 0 indicates decreasing rate with age. Significant (FDR < 0.05) and suggestive (FDR < 0.15) methylations are labelled. (B) Counts of young adult vs. later-onset BRCA cases presenting methylation-subtype pairs. (C) Counts of young adult vs. later-onset LGG cases presenting methylation-subtype pairs. (D) Percentages of young adult vs. later-onset LGG cases presenting fusion-subtype pairs. (E) Counts of young adult vs. later-onset LGG cases presenting fusion-subtype pairs.

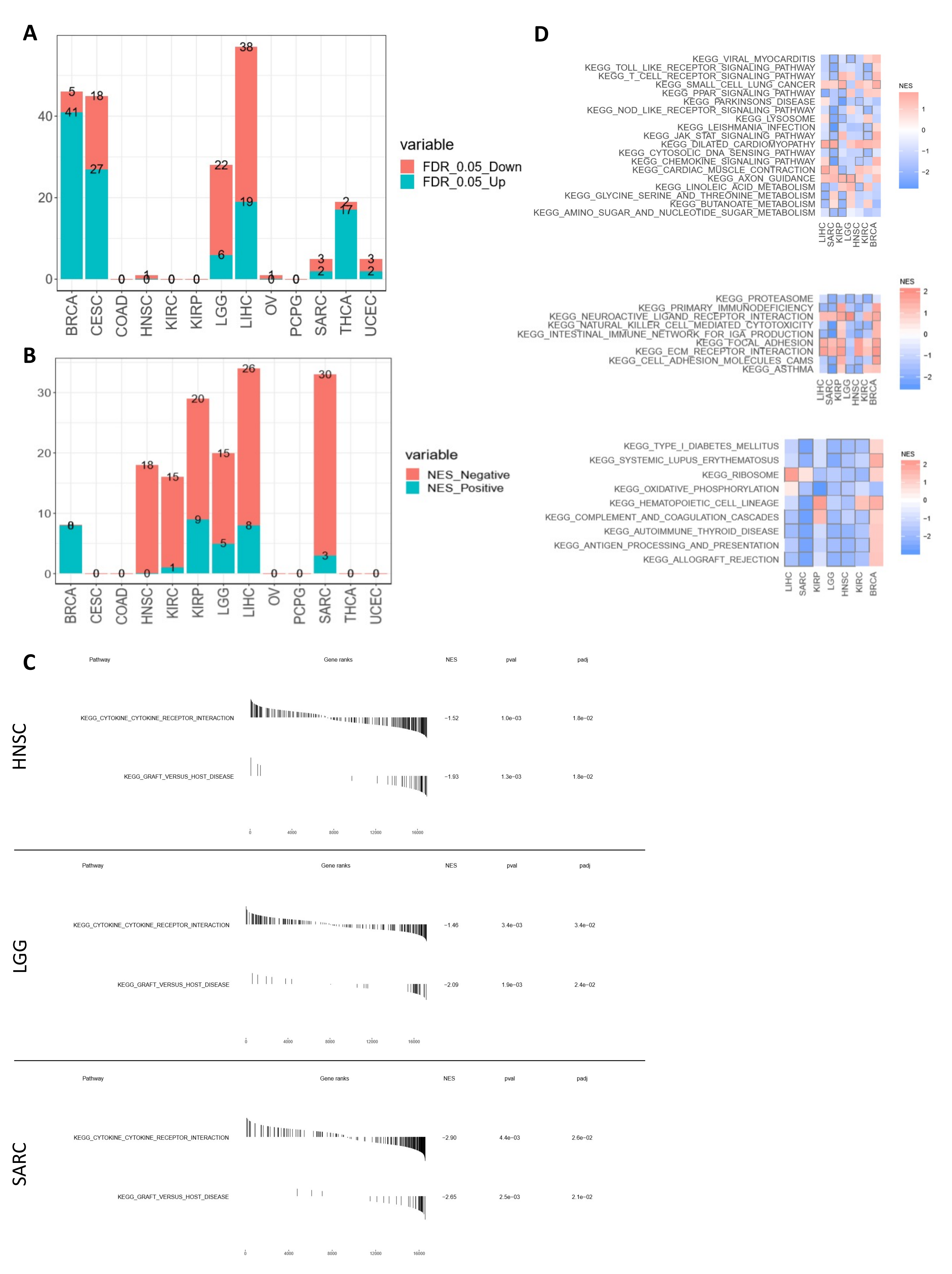

Figure S5. Further investigation of differentially expressed genes and pathways in young adult versus later-onset tumors. Related to Figure 5 (A) Number of up/down differentially expressed genes across cancers. "Up" and "Down" represent higher levels in young adult and later-onset cases, respectively. (B) Number of differentially expressed pathways with positive/negative normalized enrichment scores across cancer types. (C) Additional information regarding the enrichment of 2 immune-related pathways (cytokine-cytokine receptor interaction & graft versus host disease) with consistently lower expressions across 3 cancer types (HNSC, LGG, & SARC). (D) Normalized enrichment scores of additional pathway-cancer associations. Red and blue indicate higher levels in young adult and later-onset cases, respectively. Significant associations are pinpointed by gray boxes. Cancers are ordered by the number of significantly perturbed pathways involved, and pathways are split based on the number of involved cancers.

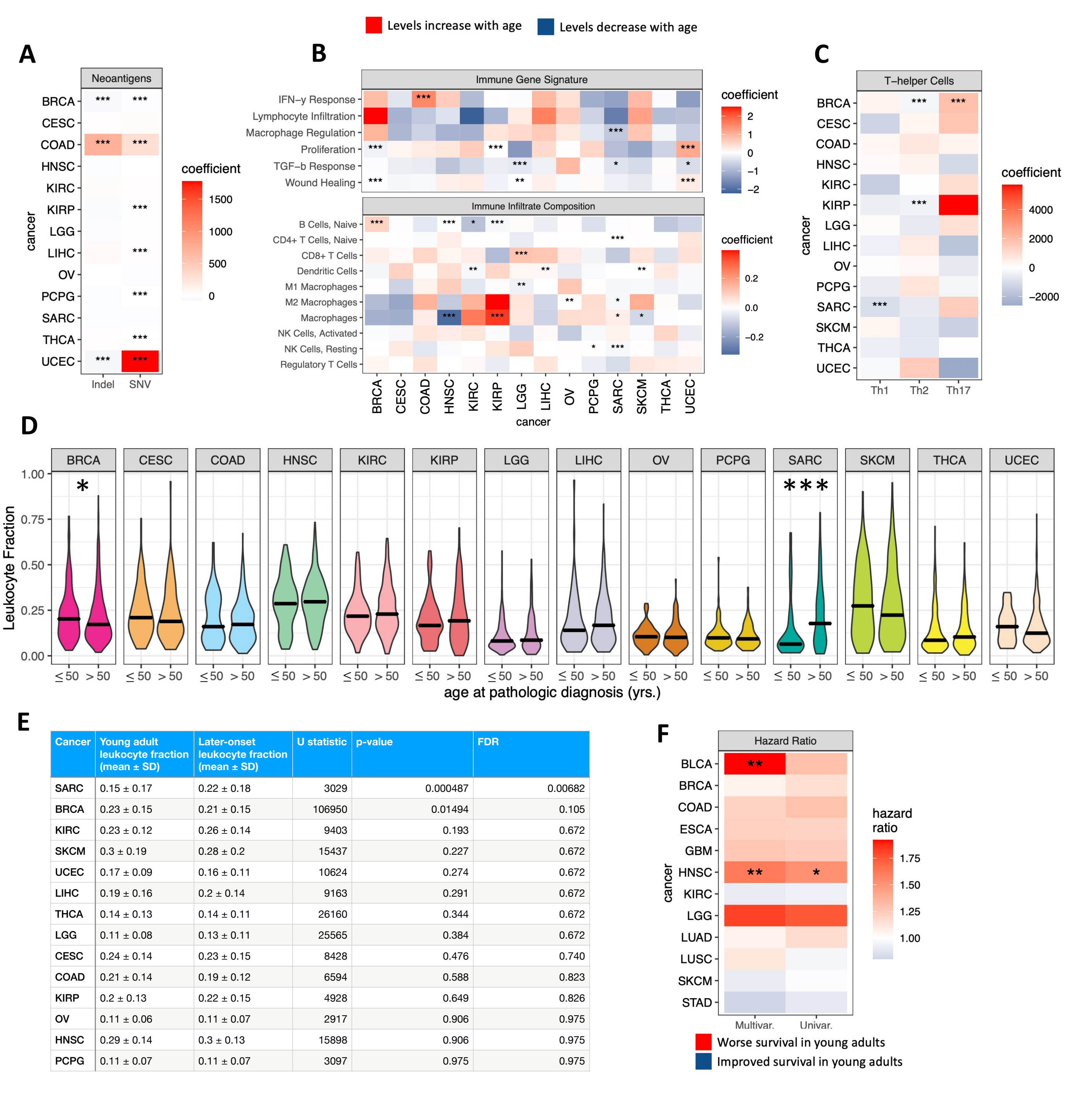

Figure S6. Additional tumor immune microenvironment investigation and survival analysis. Related to Figure 6 (A) Age-as-continuous neoantigen load analysis; red and blue indicate when neoantigen loads increase and decrease with age, respectively. * signifies an FDR ≥ 0.10 and < 0.15, ** signifies an FDR ≥ 0.05 and < 0.10, and *** signifies an FDR < 0.05. (B) Age-as-continuous immune gene signature and infiltrate analysis; red and blue indicate when immune gene signatures and infiltrates increase and decrease with age, respectively. * signifies an FDR ≥ 0.10 and < 0.15, ** signifies an FDR ≥ 0.05 and < 0.10, and *** signifies an FDR < 0.05. (C) Age-as-continuous Th cell level analysis; red and blue indicate when Th cell levels increase and decrease with age, respectively. * signifies an FDR ≥ 0.10 and < 0.15, ** signifies an FDR ≥ 0.05 and < 0.10, and *** signifies an FDR < 0.05. (D) Leukocyte fractions per sample across the 14 cancer types in young adult vs. later onset tumors; black bars designate median values. * signifies an FDR ≥ 0.10 and < 0.15, ** signifies an FDR ≥ 0.05 and < 0.10, and *** signifies an FDR < 0.05. (E) Results of Mann-Whitney U test on leukocyte fractions per sample between young adult and later onset tumors across the 14 cancer types, including mean young adult and later-onset leukocyte fractions, U statistic, and p-value. Entries are sorted by p-value in ascending order. (F) Survival analysis post-immune checkpoint inhibitor therapy; red and blue indicate worse overall survival among young adult and later-onset cases, respectively (i.e., hazard ratios > 1 and < 1, respectively). * signifies a p-value ≥ 0.10 and < 0.15, ** signifies a p-value ≥ 0.05 and < 0.10, and *** signifies an p-value < 0.05. The multivariate analysis adjusted for tumor mutational burden; the univariate did not.

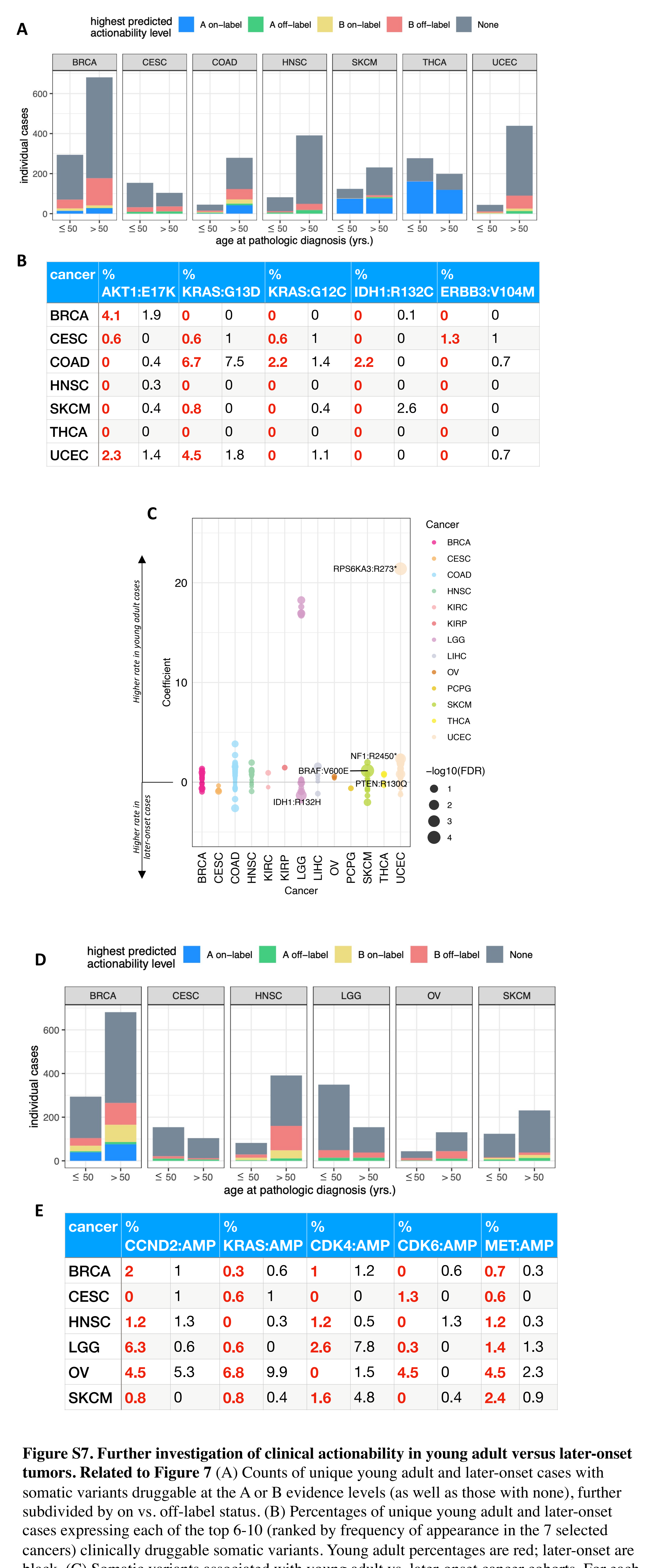

Figure S7. Further investigation of clinical actionability in young adult versus later-onset tumors. Related to Figure 7 (A) Counts of unique young adult and later-onset cases with somatic variants druggable at the A or B evidence levels (as well as those with none), further subdivided by on vs. off-label status. (B) Percentages of unique young adult and later-onset cases expressing each of the top 6-10 (ranked by frequency of appearance in the 7 selected cancers) clinically druggable somatic variants. Young adult percentages are red; later-onset are black. (C) Somatic variants associated with young adult vs. later-onset cancer cohorts. For each variant, a coefficient > 0 represents a higher rate in young adult cases, while a coefficient < 0 represents a higher rate in later-onset cases. Significant variants (FDR < 0.05) are labelled. (D) Counts of unique young adult and later-onset cases with copy number amplifications druggable at the A or B evidence levels (as well as those with none), further subdivided by on vs. off-label status. (E) Percentages of unique young adult and later-onset cases expressing each of the top 6-10 (ranked by frequency of appearance in the 6 selected cancers) clinically druggable copy number amplifications. Young adult percentages are red; later-onset are black.

Cancer	Gene-level Mutation	Coefficient	p-value	FDR
LGG	TP53	0.545776454223881	4.56172798319679E-13	3.25251205201931E-10
LGG	EGFR	-1.2570587643011	5.92112148234111E-10	1.34432545753414E-07
LGG	IDH1	-2.3330278568841	7.09007648099257E-10	1.34432545753414E-07
LGG	ATRX	0.318529877415042	7.541797798228E-10	1.34432545753414E-07
CESC	TP53	-2.37663554645042	4.11307840859638E-05	0.00509399480706927
UCEC	RPS6KA3	2.59970324406543	4.28667164690261E-05	0.00509399480706927
SKCM	NF1	-1.81576587117597	5.6018521348856E-05	0.00570588653167634
COAD	PTEN	2.18333989018961	0.000252031769201901	0.0224623314301194
UCEC	LATS1	1.85151364004937	0.000354184595426607	0.0265480994357381
UCEC	ATRX	1.2613511680316	0.000373598326030772	0.0265480994357381
HNSC	FAT1	-1.29650224769806	0.000423863610153001	0.0265480994357381
UCEC	CTNNB1	0.896709811202757	0.000446812332719294	0.0265480994357381
UCEC	PTEN	0.548508600402428	0.000504564083978032	0.0276733993751028
BRCA	GATA3	0.827344937655294	0.000702638869851232	0.0357843938717092
UCEC	FLT3	1.37603262897295	0.00098685510246205	0.0454613217821711
BRCA	CDH1	-0.734341870586539	0.00109100884550207	0.0454613217821711
UCEC	SIN3A	1.23486245479293	0.00117226557452845	0.0454613217821711
SKCM	BRAF	0.714198087717506	0.00117780870495849	0.0454613217821711
UCEC	BRD7	1.90009938671414	0.00121145177259642	0.0454613217821711
UCEC	CNBD1	1.59398383344431	0.00128271850928906	0.0457289148561551
BRCA	KMT2C	-1.00283528016813	0.00135332318803671	0.0459485444319129

Table S1. Significant age-related somatic mutations. Related to Figure 2. Gene-level somatic mutations between young adult and later-onset cancer cohorts with an FDR < 0.05, including TCGA abbreviation of the cancer type, coefficient, and p-value. Entries are sorted by p-value in ascending order.

Cancer	Copy Number Variation	Coefficient	p-value	FDR
LGG	AMP.EGFR	-1.2284621102091	4.3695732860748E-11	1.42885046454646E-08
LGG	DEL.CDKN2B	-0.725582452978816	7.0709759496738E-07	0.000115610456777167
LGG	DEL.CDKN2A	-0.678551085330074	1.63580480558435E-06	0.000178302723808694
OV	AMP.MYC	1.62169006032403	8.49484454793561E-06	0.000694453541793736
LGG	AMP.MDM4	-0.930713450346304	0.000171277767110378	0.0112015659690187
BRCA	AMP.RPTOR	1.1721594072199	0.000357848711921459	0.0195027547997195
LGG	DEL.PTEN	-18.2769041452839	0.000546288493288994	0.0255194767579287
CESC	AMP.YAP1	1.82302067684915	0.000664599265596774	0.0271654949812681
SKCM	DEL.CDKN2A	0.742727104760044	0.00165296049256719	0.0600575645632744
SKCM	DEL.CDKN2B	0.70244827798225	0.00326549831363263	0.106781794855787
UCEC	AMP.CCNE1	-16.6711935773039	0.00469327072402436	0.13359826559262
LGG	AMP.KDM5A	1.06643184771342	0.00490268864560074	0.13359826559262
LGG	AMP.KRAS	16.6996026457961	0.00652406935757926	0.14796094659789
LGG	AMP.ARRDC1	17.5087662868069	0.00652406935757968	0.14796094659789
LGG	AMP.CCND2	0.794479766684225	0.00678719938522431	0.14796094659789

Table S2. Significant and suggestive age-related copy number variations. Related to Figure 3. Copy number deletions or amplifications between young adult and later-onset cancer cohorts with an FDR < 0.15, including TCGA abbreviation of the cancer type, coefficient, and p-value. Entries are sorted by p-value in ascending order.

Cancer	Gene-level Methylation	Coefficient	p-value	FDR
LGG	EPISIL.LATS2	0.183293512496227	1.21900726523742E-10	7.92354722404321E-09
LIHC	EPISIL.CDKN2A	-1.58656768728138	4.30965950813203E-07	1.40063934014291E-05
LGG	EPISIL.NOV	-18.8270843806973	0.000150283156600603	0.00325613505967973
KIRP	EPISIL.MGA	1.98929406981366	0.00114665174816407	0.0173274310783208
SKCM	EPISIL.CDKN2A	-0.988321556242987	0.00139630347375963	0.0173274310783208
LGG	EPISIL.HES4	-0.627541559919214	0.00159945517646038	0.0173274310783208
THCA	EPISIL.CDKN2A	-0.750375975012855	0.00618188495798491	0.0574032174670028
BRCA	EPISIL.MGA	-0.913336510158911	0.00898127647253729	0.0692559008964277
BRCA	EPISIL.CDKN2A	-0.8197496835892	0.00958927858565922	0.0692559008964277
LIHC	EPISIL.TLE3	-17.6080276766965	0.0121039302517127	0.0786755466361327
KIRP	EPISIL.CDKN2A	1.43365679337096	0.0144524791050703	0.0854010128935971
BRCA	EPISIL.TCF7	-0.60875048387426	0.0188280718623801	0.101985389254559
LGG	EPISIL.TCF7	-0.475193510614517	0.0263311285537236	0.131655642768618

Table S3. Significant and suggestive age-related methylations. Related to Figure 4. Gene-level methylations between young adult and later-onset cancer cohorts with an FDR < 0.15, including TCGA abbreviation of the cancer type, coefficient, and p-value. Entries are sorted by p-value in ascending order.

Cancer	Fusion Event	Coefficient	p-value	FDR
THCA	FUSION.RET	0.946857416410663	0.0140290380326355	0.102187742275967
THCA	FUSION.NTRK3	18.2335513776732	0.019544839991687	0.102187742275967
LGG	FUSION.EGFR	-1.6179559216517	0.02189737334485	0.102187742275967

Table S4. Suggestive age-related fusions. Related to Figure 4. Fusion events between young adult and later-onset cancer cohorts with an FDR < 0.15, including TCGA abbreviation of the cancer type, coefficient, and p-value. Entries are sorted by p-value in ascending order.
